# Goal-directed control with cortical units that are gated by both top-down feedback and oscillatory coherence

**DOI:** 10.3389/fncir.2014.00094

**Published:** 2014-08-08

**Authors:** Robert R. Kerr, David B. Grayden, Doreen A. Thomas, Matthieu Gilson, Anthony N. Burkitt

**Affiliations:** ^1^NeuroEngineering Laboratory, Department of Electrical and Electronic Engineering, The University of MelbourneMelbourne, VIC, Australia; ^2^Centre for Neural Engineering, The University of MelbourneMelbourne, VIC, Australia; ^3^NICTA, Victoria Research Lab, The University of MelbourneMelbourne, VIC, Australia; ^4^Bionics InstituteEast Melbourne, VIC, Australia; ^5^Department of Mechanical Engineering, The University of MelbourneMelbourne, VIC, Australia; ^6^Laboratory for Neural Circuit Theory, RIKEN Brain Science InstituteSaitama, Japan

**Keywords:** goal-directed cognitive control, stimulus-response remapping, gain modulation, top-down feedback, neural oscillations, communication-through-coherence, prefrontal cortex, calcium spikes

## Abstract

The brain is able to flexibly select behaviors that adapt to both its environment and its present goals. This cognitive control is understood to occur within the hierarchy of the cortex and relies strongly on the prefrontal and premotor cortices, which sit at the top of this hierarchy. Pyramidal neurons, the principal neurons in the cortex, have been observed to exhibit much stronger responses when they receive inputs at their soma/basal dendrites that are coincident with inputs at their apical dendrites. This corresponds to inputs from both lower-order regions (feedforward) and higher-order regions (feedback), respectively. In addition to this, coherence between oscillations, such as gamma oscillations, in different neuronal groups has been proposed to modulate and route communication in the brain. In this paper, we develop a simple, but novel, neural mass model in which cortical units (or ensembles) exhibit gamma oscillations when they receive coherent oscillatory inputs from both feedforward and feedback connections. By forming these units into circuits that can perform logic operations, we identify the different ways in which operations can be initiated and manipulated by top-down feedback. We demonstrate that more sophisticated and flexible top-down control is possible when the gain of units is modulated by not only top-down feedback but by coherence between the activities of the oscillating units. With these types of units, it is possible to not only add units to, or remove units from, a higher-level unit's logic operation using top-down feedback, but also to modify the type of role that a unit plays in the operation. Finally, we explore how different network properties affect top-down control and processing in large networks. Based on this, we make predictions about the likely connectivities between certain brain regions that have been experimentally observed to be involved in goal-directed behavior and top-down attention.

## 1. Introduction

Our perception of the world around us and the way in which we respond to it depend on more than just the sensory information that is sent to our brains. It also depends on our recent and past experiences and on our current motivations and goals. While plasticity can make changes based upon past experiences, top-down processing allows numerous, faster changes (or switches) between stimulus-response mapping that can depend on recent events and current goals, as well as a more efficient way to allow interactions between concurrent stimuli.

The brain, in particular the cortex, exhibits a hierarchy both anatomically and functionally. Within this hierarchy, sensory information progresses “forward” through a series of regions. For example, in the visual system, stimuli cause neural activity that begins in the retina, propagates through the lateral geniculate nucleus (LGN) to the visual cortex, where it progresses through levels V1 and V2 before splitting into the dorsal (the “where” or “how” pathway) and ventral (the “what” pathway) streams and continuing further “upstream” (Goodale and Milner, 1992). In addition to this “forward” flow of information, there is much evidence that information also flows “backward” through this hierarchy. Buffalo et al. (2010) observed attentional effects that propagated from higher-order visual areas back to lower-order visual areas (i.e., V4–V2–V1).

This “backward” propagation of information, or top-down feedback, explains the observations by Womelsdorf et al. (2006, 2008) of context-dependent changes in the receptive field of neurons in visual cortical area MT. These changes included shifts of the centers of the receptive fields toward the focus of attention and narrowings of the receptive fields. Similar to this, Cohen and Newsome (2008) observed context-dependent changes in the noise correlations of MT neurons. Such top-down effects are also evident in goal-directed behavior, where the brain is able to perform fast switching between different “rules” that determine the appropriate response for a given stimulus. Wallis and Miller (2003) and Muhammad et al. (2006) showed how, during such a behavioral task, different neurons in the prefrontal (PRC), premotor (PMC), and inferior temporal (ITC) cortices and the striatum (STR) responded selectively to either the task rule (desired stimulus-response mapping), the behavioral response carried out, the visual stimulus being remembered, or whether or not the subsequent stimulus matched this remembered stimulus.

In order to perform tasks such as top-down attention and goal-directed behavior, the functional connectivity of cortical networks must be rapidly and flexibly modifiable. Haider and McCormick (2009) reviewed the evidence for neural activity producing this rapid modulation in the functional connectivity. In this study, we focus on two different mechanisms for rapidly rearranging the functional connectivity of cortical networks: *gain modulation* and *communication-through-coherence*.

Gain modulation is where one type of input modulates the gain or sensitivity of a neuron to another type of input (Salinas and Sejnowski, 2001). Top-down gain modulation of neuronal responses that is dependent on contextual information or a different type of stimulus has been observed; however, the neuronal mechanisms underlying it have not been well understood. Larkum and colleagues found that pyramidal neurons exhibit a much stronger response when they receive inputs from both feedforward and feedback connections (Larkum et al., 1999; Larkum, 2013), which tend to be targeted to the cell's soma and basal dendrites and to the cell's apical dendrites, respectively (Felleman and Van Essen, 1991). This non-linearity is due to interactions between the sodium and calcium spike initiation zones of pyramidal neurons, which are located at the soma and apical branch, respectively. This suggests that feedback connections to pyramidal neurons from higher-order regions can be thought of as modulating the gain of the neurons they target. While gain modulation provides a means for top-down processing or control, this has not been fully explored and there are limitations to the influence that is possible.

Synchronization and oscillations are ubiquitous in the cortex. Gamma oscillations, in particular, have been shown to be important in higher brain functions (Bartos et al., 2007; Fries et al., 2007), such as (selective) attention (Womelsdorf and Fries, 2007) and top-down processing (Engel et al., 2001). Communication-through-coherence (CTC) proposes that coherence between the oscillations of different neuronal groups modulates and routes communication through the brain (Fries, 2005). Supporting this hypothesis, synchronization and phase relations have been observed to govern interactions between neuronal groups (Womelsdorf et al., 2007). Gregoriou et al. (2009) showed that the prefrontal cortex and V4 exhibited long-range coupling of activity at gamma frequencies, initiated in the prefrontal cortex. There is much evidence suggesting that gamma (and beta) oscillations are involved in top-down and bottom-up interactions between the prefrontal and visual cortices (Benchenane et al., 2011).

Theoretical work has also shown how synchrony or coherence can act as a modulator of the gain of pyramidal neurons (Tiesinga et al., 2004; Börgers et al., 2005; Mishra et al., 2006; Tiesinga et al., 2008; Tiesinga and Sejnowski, 2009) and has also examined how top-down gain modulation can enable networks of neurons to perform fast stimulus-response remappings (Salinas, 2004). However, this situation has not been explored theoretically with neurons whose gain is simultaneously modulated by two different mechanisms: top-down (apical-targeted) feedback and oscillatory coherence. Furthermore, there has not been sufficient attention paid to understanding how gain modulation behaves and is controlled in hierarchical networks with several levels/layers.

In this paper, we develop a simple neural mass model in which units exhibit gamma oscillations when they receive coherent oscillatory inputs to both the apical dendrites (feedback) and the soma/basal dendrites (feedforward). In this way, activity is modulated by two different mechanisms: apical-targeted, top-down feedback and oscillatory coherence. We explore how these units can be formed into circuits to perform the same types of logic operations (e.g., “AND,” “OR,” and “NOT”) considered by Vogels and Abbott (2005). Similar to previous studies involving gain modulated units (Salinas, 2004), we consider how these logic operations can be initiated and controlled (i.e., altered) by top-down feedback. However, unlike previous studies, we identify the different ways in which this top-down control can be implemented in hierarchical networks. In the same way that top-down gain modulation can strengthen or weaken the activity of neurons, we show that units can be added to or removed from a higher-level unit's logic operation by altering the feedback activity that this higher-level unit is given. Furthermore, by modeling units as oscillating with a particular phase, we show that it is possible for feedback to modify the type of role that a unit has in the operation. This is not possible with top-down gain modulation alone and requires the additional coherence modulation. We explore how different network properties affect top-down control and processing in the networks, and make predictions about the likely connectivities between the different brain regions that have been experimentally observed to be involved in goal-directed behavior and top-down attention.

## 2. Materials and methods

### 2.1. Cortical unit model

**Figure 1:**
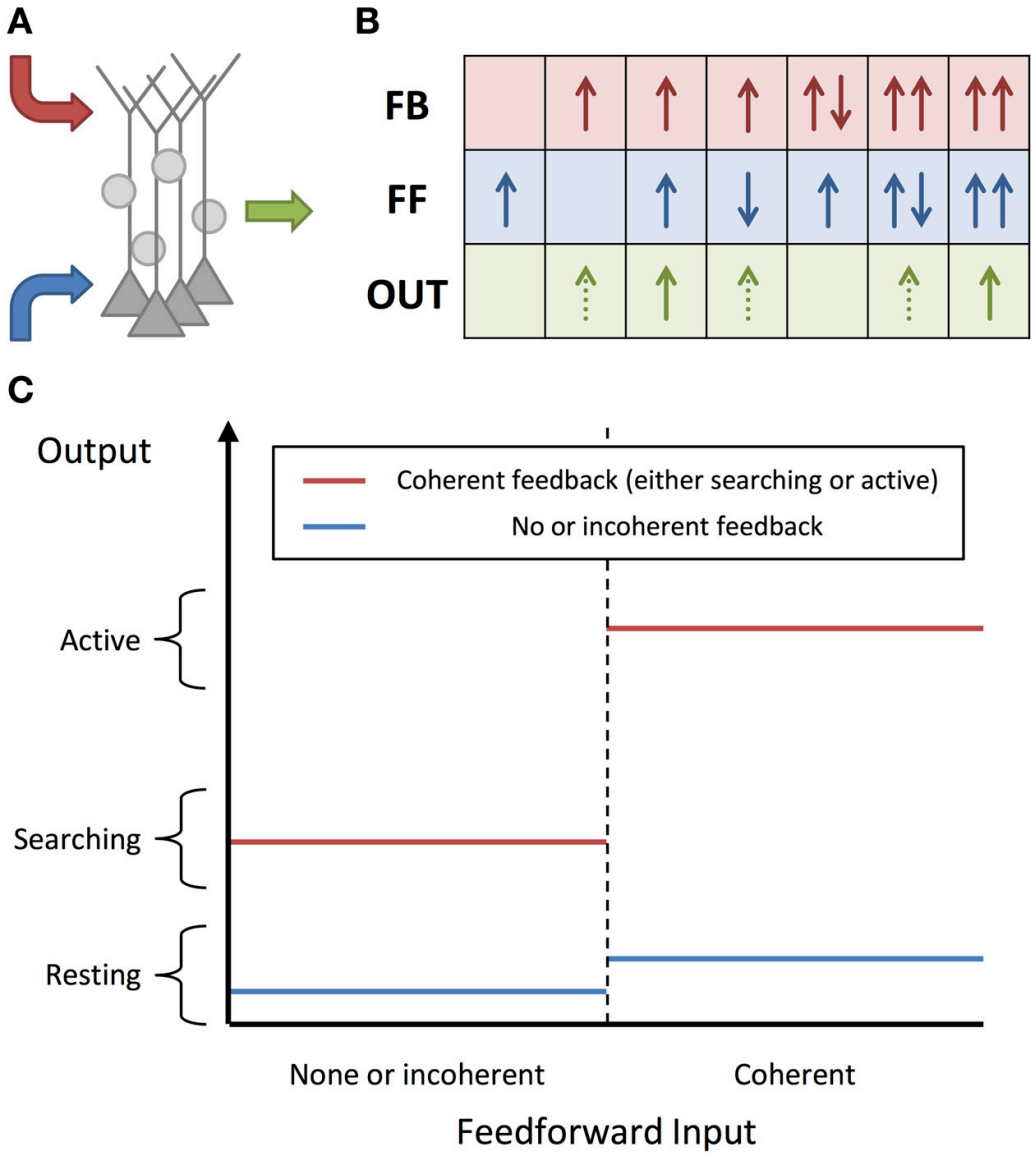
**Diagram of Model. (A)** A cortical unit, composed of pyramidal neurons and inhibitory interneurons, exhibits activity (green) based on the feedforward, basal/soma-targeted (blue) and feedback, apical-target (red) inputs it receives. **(B)** Table describing how the unit activity depends on these inputs, as described by Equations (2) and (3). The inputs and outputs are shown by solid and dashed arrows, which correspond to active and searching inputs/outputs, respectively. The direction of each arrow indicates the phase of gamma oscillations (active) or the timing of sporadic, feedback-propagating bursts (searching). The different rows correspond to feedforward and feedback inputs, and unit output, respectively. Multiple feedforward or feedback arrows indicate multiple inputs of these types. Note that the same effects are achieved with sporadic, bursting feedback inputs (but not so for feedforward inputs). **(C)** Modulating effect of feedback on a unit's responsiveness to feedforward input, as described by Equation (2). Without feedback, the unit will remain in the resting state, regardless of the feedforward input. Coherent feedforward input must be coherent within itself but also with any feedback activity.

We model the cortex as being composed of a network of small units of pyramidal neurons and inhibitory interneurons (Figure 1A). These units are modeled as neural masses and the individual neurons are not explicitly modeled. The units receive two types of inputs: feedforward inputs to the soma and basal dendrites (blue) and feedback inputs to the apical dendrites (red). As proposed by Larkum (2013) for individual pyramidal neurons, we hypothesize that these units are associative and generate much stronger output when they are activated simultaneously by both of these types of inputs. We further hypothesize that these units can only be activated by inputs with gamma oscillations. Importantly, it is an assumption of the model that the input activity, and the activity elicited in the units, is oscillatory. While inputs and units in the model are not actually composed of networks of neurons that generate these oscillations, the oscillations do represent fluctuations of the instantaneous spiking rate of neural populations and we assume that in the brain they would arise due to the reciprocal excitation and inhibition within the population. In addition to receiving both feedforward and feedback input, activation of units requires that these inputs are in phase, or coherent (Figure 1B). The requirement for units to receive both feedforward and feedback activity in order to become active can be thought of as binary gain modulation or a gating of the unit's activity (see Figure 1C).

Our model has a coarse time-step equal to half the period of a typical gamma oscillation (about 7–10 ms). At a given time-step, the state of each unit, *s_i, t_*, takes on one of three possible values:

- **Resting:** units exhibit insufficient activity to affect other units;
- **Searching:** units exhibit strong but sporadic bursts of activity that can propagate and affect other units via feedback connections;
- **Active:** units exhibit strong, gamma-frequency activity that affect other units via feedforward or feedback connections.

The activity of units is confined to gamma oscillations and, consequently, units can only be active every second time-step and, therefore, must have one of two possible phases. In other words, oscillations in units in the model are simply active states that occur on alternating time-steps. We intentionally restricted oscillations in the model to having only two possible phases in order to keep the model sufficiently simple so that networks of units could be analyzed and their function could be understood. While, the resting and active states correspond to the on and off states of binary models, the searching state represents a novel type of state, where units have not been fully activated but are still able to pass down the feedback they receive to lower-levels. We refer to this as the searching state as it can be thought of as searching lower-level units that the unit sends feedback connections to. It is then able to ignite activity in units that are receiving feedforward activity. In this way, top-down feedback allows feedforward activity to propagate to higher-levels, which it would be unable to do otherwise.

While individual neurons are able to fire in response to only feedforward or feedback input, we are hypothesizing that, in higher-level areas of the cortex, groups/ensembles of neurons (units) are generally only able to be activated to a sufficient degree when the neurons in these groups receive both feedforward and feedback activity. Active units in our model exhibit strong gamma oscillations and can significantly affect the firing of other units that they are connected to. However, within a resting unit, the neurons are still assumed to be firing (perhaps even as gamma oscillations), although we have assumed that the firing is at a lower rate and is not sufficient to significantly affect the activity of other units that they are connected to. In this way, we are still modeling feedback as modulating the activity of groups of neurons (as illustrated by Figure 1C). However, because we only consider three different levels of activity (resting, searching, and active), the feedback modulation effectively becomes a gating of unit activity.

The state of each unit is determined by the inputs that it received from other units in the previous and current time-steps. These inputs come from other units via connections with short (~0 ms, negligible) or long time lags (~7–10 ms, one time-step). We denote the sets of short connections into unit *i* as${\hat{F}}_{i}$ and ${\hat{B}}_{i}$ (feedforward and feedback, respectively) and the sets of long connections into unit *i* as *F_i_* and *B_i_* (feedforward and feedback, respectively). The presence of feedforward and feedback inputs into each unit are summarized by the Boolean expressions

(1) $$\begin{array}{l} f_{i,t}=\{\underset{j\in {\hat{F}}_{i}}{\cup }[s_{j,t}\text{ is active}]\}\cup \{\underset{j\in {\bar{F}}_{i}}{\cup }[s_{j,t-1}\text{ is active}]\},\\ b_{i,t}=\{\underset{j\in {\hat{B}}_{i}}{\cup }[s_{j,t}\text{ is active or searching}]\}\cup \\ \;\{\underset{j\in {\bar{B}}_{i}}{\cup }[s_{j,t-1}\text{ is active or searching}]\}, \end{array}$$

respectively. As mentioned above, the activity of units can be thought of as having a phase. In this view, activity arriving at a target unit will have the same phase as the source unit for connections with short time delays or the opposite phase to the source unit for connections with long time delays.

The state of unit *i* is given by

(2) $$s_{i,t}\;=\{\begin{array}{ll} \text{resting } & \text{if }\neg b_{i,t}^{*}\\ \text{searching } & \text{if }b_{i,t}^{*}\cap \neg f_{i,t}^{*}\\ \text{active } & \text{if }b_{i,t}^{*}\cap f_{i,t}^{*}, \end{array}$$

where f^*^_i, t_ and b^*^_i, t_ are Boolean expressions for whether the basal/soma and apical compartments, respectively, of the pyramidal neurons in unit *i* receive coherent inputs and become activated in time-step *t*. This is illustrated in Figure 1C and shows that units are only activated if both of these compartments are activated in its pyramidal neurons. The unit is in the searching state if the apical compartment is activated but the soma/basal compartment is not. The unit is in the resting state if the apical compartment is not activated.

Gamma oscillations are generated in the cortex through activation of either networks of inhibitory neurons, via the interneuron gamma (ING) mechanism, or reciprocally connected networks of excitatory and inhibitory neurons, via the pyramidal-interneuron gamma (PING) mechanism (Whittington et al., 2000; Brunel and Wang, 2003; Tiesinga and Sejnowski, 2009). Given the role that inhibitory neurons therefore have in producing gamma oscillations, we have assumed that the (basal/soma or apical) compartments of a unit are shut down by the inhibitory neurons if they receive non-coherent inputs. Put another way, the incoherent inputs interfere with the rhythmic interaction between the excitatory and inhibitory neurons in the unit. This is described by

(3) $$\begin{array}{l} f_{i,t}^{*}=f_{i,t}\cap \neg f_{i,t-1}\cap \neg f_{i,t-3},\\ b_{i,t}^{*}=b_{i,t}\cap \neg b_{i,t-1}\cap \neg b_{i,t-3}. \end{array}$$

The compartments cannot be activated in consecutive time-steps as the inhibitory population constrains the activity to gamma oscillations. Activity in one time-step not only prohibits activity in the next time-step, but also two time-steps after that. In this way, there is a phase given to the activity of units. By considering the model in terms of phases, the input/output relations in Figure 1B present another perspective. Provided there is coherent feedback inputs, there is at least the sporadic, searching signal (green dotted arrows) that can propagate down to lower levels. If, additionally, there are coherent feedforward inputs that are also in phase with the feedback, then the unit becomes active and exhibits strong gamma oscillations (green solid arrows).

### 2.2. Cortical networks

**Figure 2:**
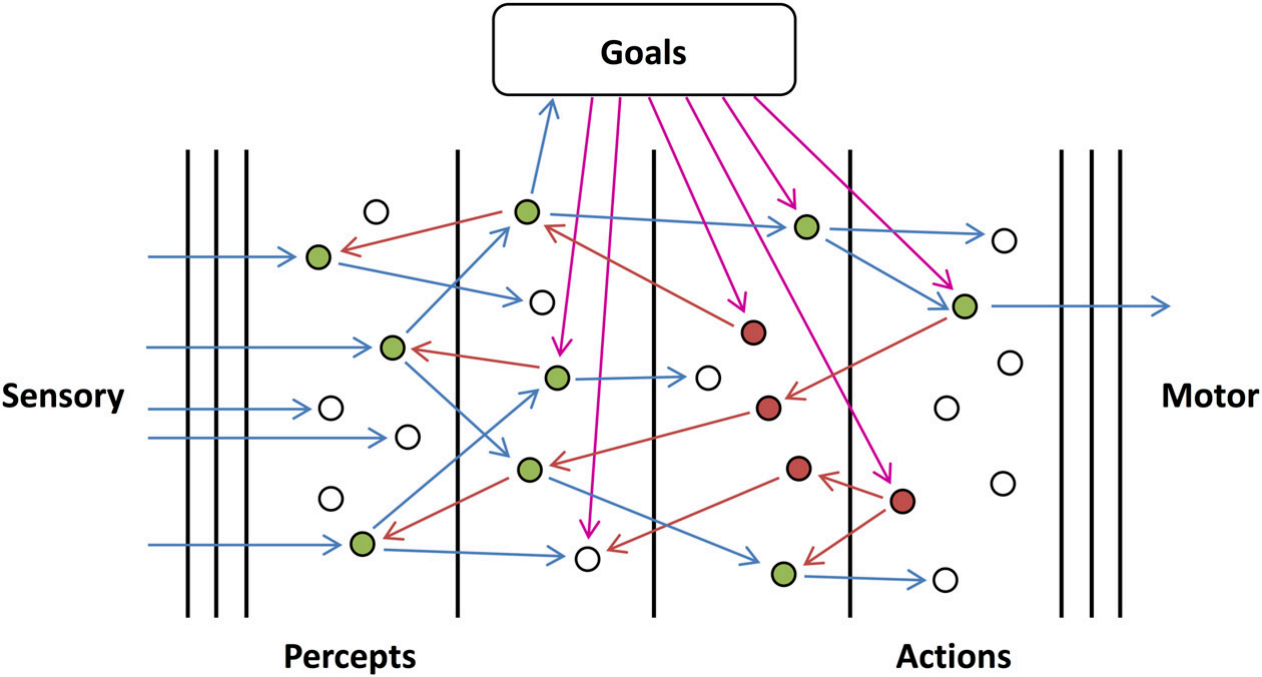
**Goal-directed Network**. An illustration of the proposed cortical architecture. Sensory, feedforward input (left) is mapped to percepts, actions, and finally motor responses (right), and this mapping is controlled by goal-dependent feedback (top). In the diagram, blue, red, and magenta arrows correspond to feedforward, internal feedback and external feedback (feedback corresponding to the goals of the system) connections, respectively. It should be noted that only the connections from active or searching units have been shown and they would exist other connections which have not been shown. White, green, and red units correspond to resting, active, and searching units, respectively.

In this paper, we consider that the previously defined cortical units are organized into architectures similar to that presented in Figure 2. Here, the system receives sensory inputs (left) and produces motor outputs (right). Units in the system represent abstract concepts, such as percepts and actions, that depend on the sensory inputs and determine the behavior, respectively. In Figure 2, we divided the architecture into levels (using vertical black lines). These levels embody a hierarchy in the processing of information. Feedforward connections are made from units in lower levels to units in higher levels while the reverse is true for feedback connections. Here, the number of levels depicted is arbitrary and for illustrative purposes; the actual number of levels is most likely much greater. Similarly, the multiple vertical lines between the sensory and the percepts, and between the actions and motor, are only intended to indicate that there would be a number of levels of processing (e.g., for the visual pathway: those in the retina, LGN, V1, etc.) in between. The levels aim to convey the idealized version of the functional architecture that we consider in this paper.

Units in a network require feedback in order to become activated. For units in the networks that we are considering, this feedback must arrive from an external source, otherwise no units can become activated regardless of the sensory, feedforward inputs that they receive. We assume this external feedback arrives from higher-level networks or areas of the brain. We rely on the assumption that there exists at least one high-level region that provides this feedback to the rest of the brain without receiving feedback itself. This feedback would be dependent on the goals, motivation, and state of the system (working memory), and would control the way in which the network causes percepts to lead to actions. While these goals must be reasonably persistent, feedforward activity of certain percepts would assumedly have the ability to affect these goals; however, we are not going to consider how these goals persist or change in this paper.

In order for arbitrary mappings from percepts to actions to be made, units receive feedback activity and perform logic operations on their inputs. In a sense, they are “asking” questions or testing hypotheses regarding the state of these inputs. Higher-level units will in turn use the outputs of these lower-level units as inputs. As illustrated in Figure 2, lower-level units, or groups of units, represent different percepts formed about the sensory information received while higher-level units, or groups of units, begin to more appropriately resemble different courses of action for the system to perform. The logic operations that each unit performs can be thought of as hypotheses about the state of the external world and hypotheses about what (if any) actions should be carried out.

We consider networks of units to be composed of smaller subnetworks. For the subnetwork being considered, we refer to the lowest-level units as being the inputs and the highest-level units as being the output units. These units are identical in terms of how they are modeled but play different roles in the larger network. We assume that only input units receive feedforward activity from lower-level units outside the subnetwork and only output units send feedforward activity to higher-level units outside the subnetwork (it is only their activity that matters to higher-level areas).

Just as the units in our model require feedback to become active, networks (and subnetworks) of these units require external feedback in order for any of their units to become active. For a given subnetwork, external feedback can not only be to output units, it can also be to input units and intermediate units (units that are neither input nor output units). We refer to external feedback that arrives at output units as *initiating* feedback (as it initiates the output units) and external feedback that arrives at lower-level units in the subnetwork as *orchestrating* feedback (as it orchestrates or manipulates the operations performed by the output units). These two types of feedback are not different in the way that they affect units but are distinguished by the different functional roles they play. Importantly, their roles are specified relevant to the subnetwork being considered. For example, orchestrating feedback for one subnetwork may be initiating feedback for another subnetwork. In the network in Figure 2, external feedback is represented by the dotted red arrows that are dependent on the current goals.

### 2.3. Logical operations

**Figure 3:**
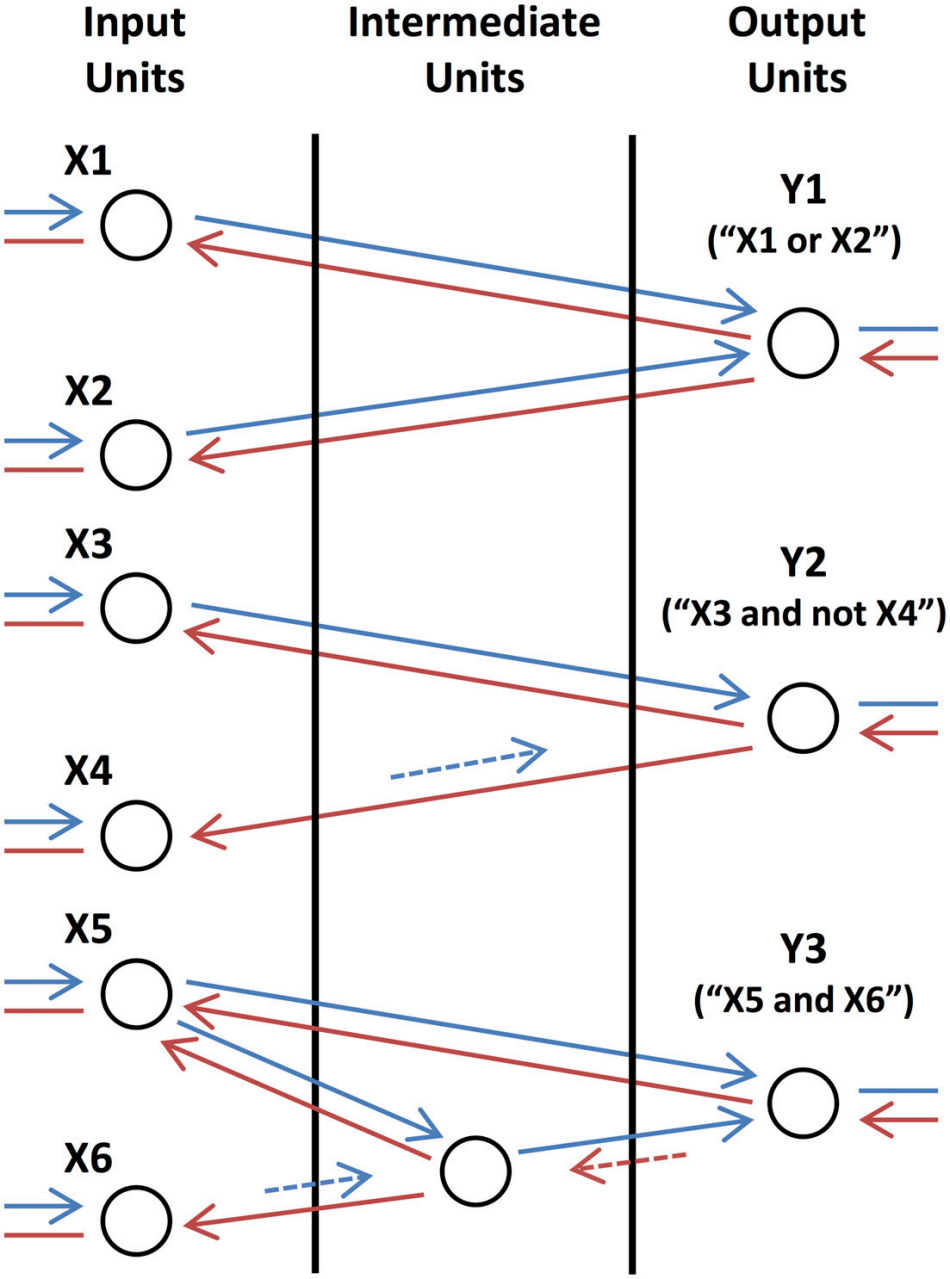
**Basic Logic Motifs**. From top to bottom, motifs in which the output units compute the operations “X1 or X2,” “X3 and not X4,” and “X5 and X6,” respectively. Arrows correspond to feedforward (blue) and feedback (red) connections with short (dashed and shorter length) and long (solid and longer length) time lags. Arrows are also shown connecting the input units to lower-level units (not shown) and connecting the output units to higher-level units (not shown) as these motifs function as circuits in a larger network.

We consider examples of simple subnetworks, or motifs, in order to illustrate the functional roles of different types of connections. Shown in Figure 3, these motifs perform simple logic operations when initiated by external feedback. The output units, Y1, Y2, and Y3, send feedback activity to input units, X1, X2, X3, X4, X5, and X6, and become activated if they receive feedforward activity in return. Y1 (“X1 or X2”) becomes activated if either X1 or X2 receives coherent, feedforward input, which, because of the long time lag of the connections, must be out-of-phase with the activity of Y1 so that they can provide returning feedforward activity that is coherent with Y1's activity. Y2 (“X3 and not X4”) becomes activated only if X3 receives coherent, feedforward input and X4 (which makes a short feedforward connection onto Y2) does not, as activity from this unit would arrive out-of-phase with Y2's activity. In the last motif, the unit in the intermediate layer performs the same operation (“AND NOT”) on its inputs as Y2. Y3 (“X5 and X6”), in turn, also performs the same (“AND NOT”) operation as Y2 except that the intermediate unit is initiated in phase with Y3 and so the time lags of the connections between them are reversed.

Generally, we denote whether or not the output unit *i* is activated by *y_i_*, which can either be true or false. This is determined by the operation that the unit performs, which is given by the binary function

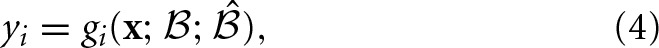



where the binary vector **x** denotes whether or not each of the input units are receiving feedforward inputs of the appropriate phase, 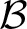 denotes the set of external feedback (and phase of the feedback) that each output unit receives (initiating feedback), and 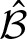 denotes the set of external feedback (and phase of the feedback) that each lower-level unit receives (orchestrating feedback). We refer to 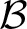
^ϕ^ and 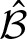
^ϕ^ as the empty feedback sets, where there is no initiating and orchestrating feedback, respectively, and to 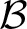
^*i*^ as the set in which only output unit *i* receives initiating feedback. Unless 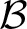 includes feedback to unit *i*, *g_i_*(**x**;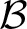;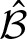) = 0 for all inputs. As we have motifs that can perform the “OR” and “NOT” operations, we can compose these together to form networks to perform arbitrary logic operations.

## 3. Results

The brain is able to perform arbitrary mappings from different stimuli to different behavioral responses and to rapidly modify its mappings based on current motivations and goals. In order to understand how this occurs, we have proposed a model of groups or ensembles of neurons that when connected into networks can produce arbitrary mappings. We explore the different ways in which they can be controlled by top-down feedback from higher brain regions and how this depends on the connectivity of these networks.

### 3.1. Top-down processing

Goals influence the operations of units through providing external feedback to the network. For a given set of output units, this feedback can be divided into initiating feedback, which targets the output units, and orchestrating feedback, which targets lower-level units. Where the goals send initiating feedback to multiple output units, the operations performed by these units may be either *non-interacting* or *interacting*. Where the goals send orchestrating feedback to units in the network, the operations may be *orchestrated*. Each of these types of operations shall now be described in turn.

#### 3.1.1. Non-interacting operations

Operations performed by output units are non-interacting if the units perform the same operations when they are initiated together as they did when initiated separately. This means that the operations can be performed in parallel without affecting each other. Using functional notation, we say that the operation of unit *j* does not interact with the operation of unit *i* for orchestrating feedback to the network 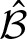 if, for all sets of inputs **x**,

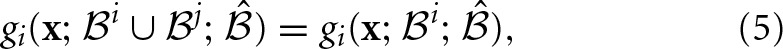



where 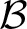
^*i*^ denotes the set with initiating feedback to only output unit *i* and 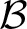
^*i*^ ∪ 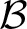
^*j*^ denotes the union of the sets 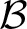
^*i*^ and 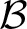
^*j*^ with initiating feedback to only output units *i* and *j*.

#### 3.1.2. Interacting operations

Interactions occur when a unit's operation is modified by other units being initiated alongside it (i.e., when output units are initiated together). The number of operations that can be performed in parallel is limited by the number of interactions that occur. In the most extreme case, there is only one information channel due to the dependencies between the units and collectively the output units only perform a single, more complex operation. Interactions allow top-down processing, as feedback into one unit can affect the operations performed by other units in the network. Using functional notation as for non-interacting operations, we say that the operation of unit *j* interacts with the operation of unit *i* for orchestrating feedback to the network 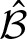 if there exists a set of inputs **x** for which

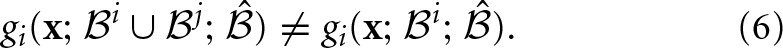



**Figure 4:**
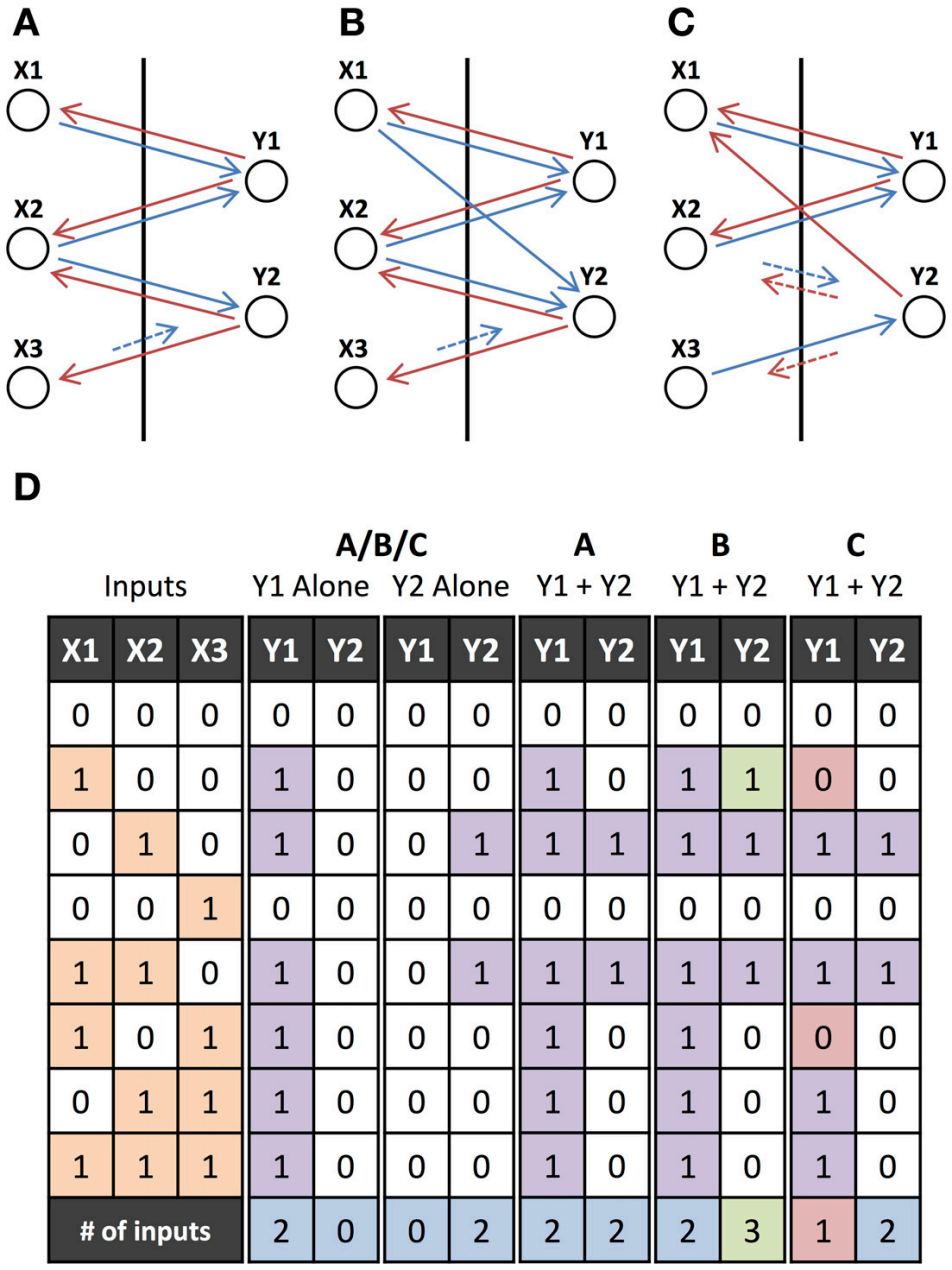
**Non-interacting and Interacting Operations. (A)** Two output units, Y1 and Y2, which individually perform operations “X1 or X2” and “X2 and not X3,” respectively. Feedforward connections to input units from lower-level units and feedforward connections from output units to higher-level units (as shown in Figure 3) have been omitted. **(B)** Same as **(A)** but with an additional feedforward connection which does not change the individual operations but introduces interactions when they are initiated together. **(C)** Same as **(A)** but Y2 instead needs to be initiated in phase with the input units for it to perform the same operation. There is also an additional feedback connection that, similar to the additional connection in **(B)**, does not change the individual operations but introduces interactions. **(D)** Input-output table for the networks in **(A–C)**. The input units (or cues), X1, X2, and X3, either receive feedforward input (1) or not (0), and the output units, Y1 and Y2, are either activated (1) or not (0) for each of the networks initiated with external feedback to only Y1, only Y2, or to both Y1 and Y2. Green (red) outputs are ones that are activated (not activated) when the units are initiated together but were not activated (activated) when the units were initiated separately. The final row indicates the number of inputs that the output unit's operation depends upon (relevant inputs), where green (red) indicates that the number has increased (decreased) from being initiated separately to being initiated together.

Figure 4 illustrates the contrast between interacting and non-interacting operations. It also demonstrates how small changes to a network with non-interacting operations cause it's operations to interact. Figure 4A shows a network where two operations have overlapping inputs but no interactions occur. Figures 4B,C provide examples of networks with overlapping motifs where the same operations are performed when the outputs are initiated separately but interactions occur when they are initiated together. These interactions are evident in the table in Figure 4D, where there exist inputs for which a different output is produced depending on whether the two hypotheses are initiated separately or together. The last row in Figure 4D contains the number of inputs that are involved in the operations performed. Interactions can cause this to either increase or decrease.

#### 3.1.3. Orchestrated operations

Orchestrating feedback (from an external source) can alter the operations that an output unit in a subnetwork performs. In this case, the feedback manipulates or controls the operations that are performed. We found dependencies between interactions that occur between units and the level and type of control that is possible by orchestrating feedback. We say that the operation of unit *i* has been orchestrated if the orchestrating feedback 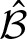 causes there to exist a set of inputs **x** for which

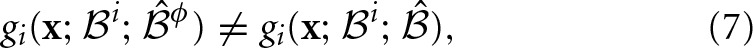



where 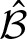
^ϕ^ denotes the empty set where there is no orchestrating feedback to the network.

**Figure 5:**
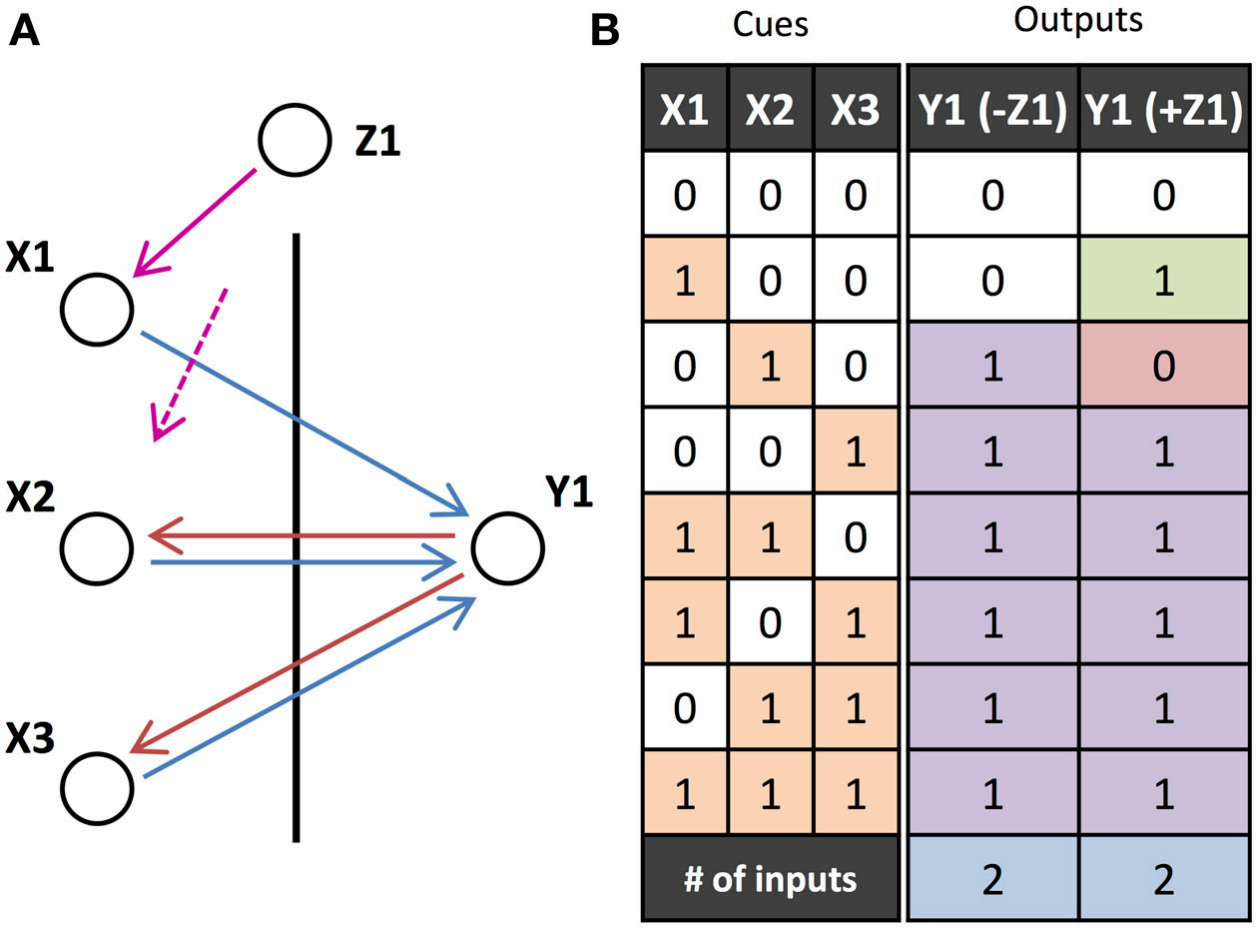
**Orchestrated Operations. (A)** Motif performing an “OR” operation over the two input units X2 and X3 has the input unit X2 removed and X1 added by orchestrating feedback (magenta arrows) from unit Z1 which, like Y1, is initiated out-of-phase with the inputs. **(B)** Input-output table for the network in A. The input units (or cues), X1, X2, and X3, either receive feedforward input (1) or not (0), and the output unit Y1 is either activated (1) or not (0), in the cases where there is feedback or not from Z1. The final row indicates the number of inputs that the output unit's operation depends on (relevant inputs).

Figure 5 shows the mechanisms by which external feedback *adds* or *removes* units from an operation. Without any external feedback, the operation performed is “X2 or X3.” By adding the external feedback, X2 is removed from the operation and X1 is added, making the operation “X1 or X3.” This could be orchestrating feedback from outside the network (as we have shown) but it could also be feedback from another output unit that is initiated with Y1.

### 3.2. Goal-directed behavior

**Figure 6:**
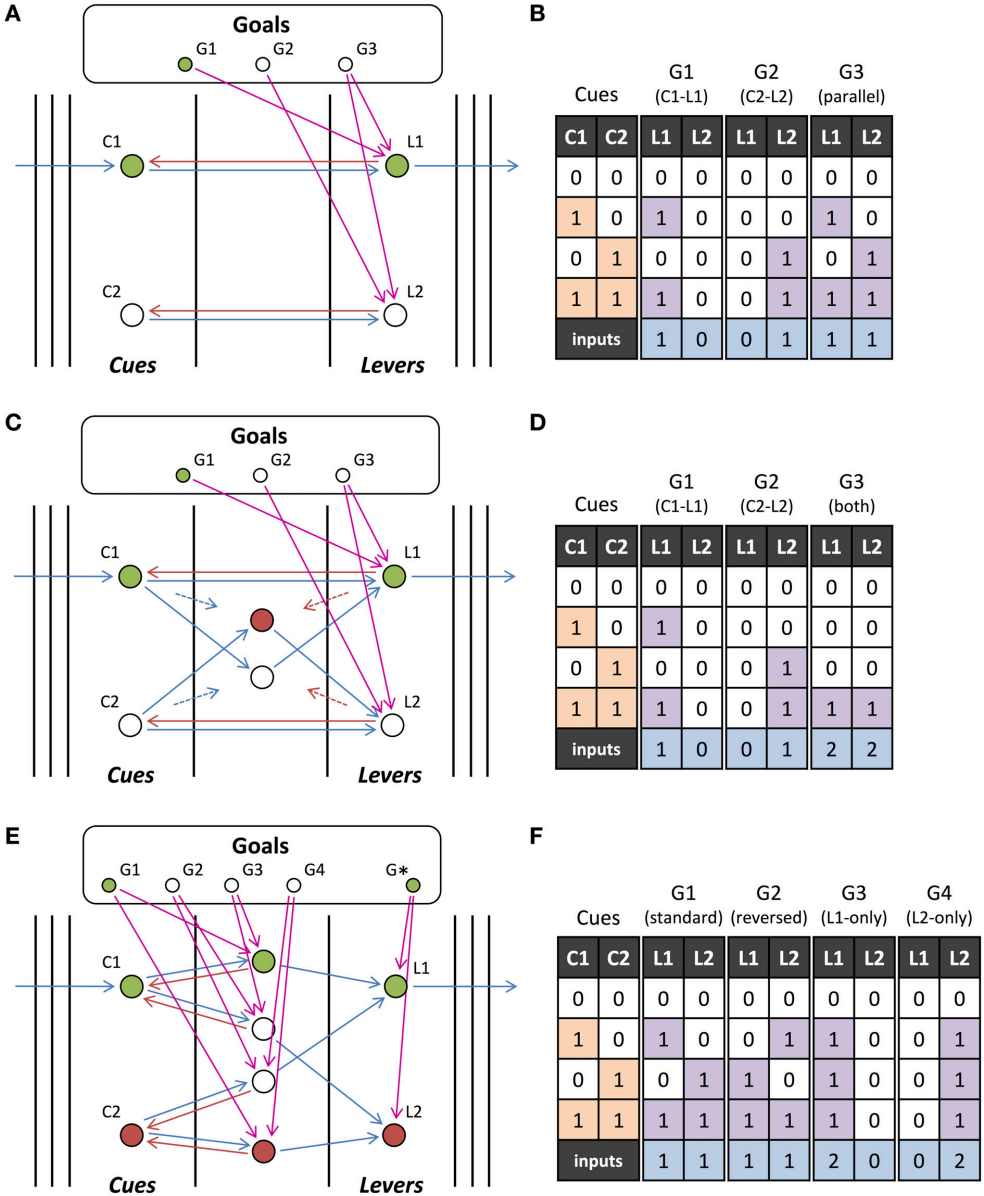
**Stimulus-response Tasks. (A)** An example of how the cortical architecture would be utilized for a stimulus-response task where a subject pulls one of two levers when presented with one of two sensory cues. The task switches between one of three “goals”: lever L1 should be pulled for cue C1 and cue C2 ignored (G1), L2 for C2 and C1 ignored (G2), and L1 for C1 and L2 for C2 (G3). Network activity is shown for when G1 is active. Similarly, the arrows to the cue units from the left and those leaving the lever units depict the inputs and outputs of the network (i.e., only “active” connections) for a particular set of inputs. **(B)** Input-output tables for the network shown in **(A)** for the three different goals. The final row indicates the number of inputs that the output unit's operation depends on (the relevant inputs). **(C)** Same as **(A)** but the third task (G3) now involves pulling both levers if and only if both cues occur together. Note that the feedback from the three goals is the same as in **(A)** but there is an extra layer in the network. **(D)** Same as **(B)** but for the network in **(C)**. **(E)** Similar to **(A,C)** but with four different goals: L1 should be pulled for C1 and L2 for C2 (G1), L2 for C1 and L1 for C2 (G2), L1 for either C1 or C2 (G3), and L2 either C1 or C2 (G4). G^*^ is not actually one of the four goals but instead always provides feedback (each of the goals could instead provide this feedback). The feedback from the goals is no longer only to output units. **(F)** Same as **(B,D)** but for the network in **(E)**.

Figure 6 shows examples of networks that can perform possible stimulus-response experiments, where switching between different rules or goals, is required. Each of the networks in Figures 6A–C, has two different percepts (stimulus cues) as inputs, two different actions (levers to pull) as outputs, and a number of different goals, rules, or stimulus-response mappings that direct how these inputs lead to different outputs. The tables correspond to the binary functions, *g_i_*(**x**;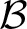;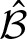), where *i* corresponds to the two levers, or output units, **x** denotes the cues that are present, and 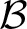 and 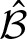 denote the initiating and orchestrating feedback, respectively, from the goal units.

In Figures 6A,C, each of the goals only sends initiating feedback to the network. In Figure 6A, there are three different goals: G1, to pull lever L1 when cue C1 is presented (and ignore C2); G2: to pull lever L2 when cue C2 is presented (and ignore C1); and G3, to pull lever L1 when cue C1 is presented and pull lever L2 when cue C2 is presented (perform both goals in parallel). These goals control the network through feedback to the two units corresponding to the two lever actions. The inputs of the output units do not overlap, so trivially their operations (performed separately by G1 and G2) are non-interacting and so can be performed in parallel (by G3). Figure 6B shows the input-output table for this.

In Figure 6C, there are also three different goals, the first two of which are the same as the first two in the network in Figure 6A. The third goal is to pull levers L1 and L2 when both cues C1 and C2 are presented (and ignore both C1 and C2 presented alone). The same feedback from the three goals as in Figure 6A is used to control the network, but different behavior (Figure 6D) arises due to differences in the networks. In Figure 6B, we see that the conditions for L1 and L2 remain the same regardless of whether they are included in the task or not (i.e., the logic operations can be performed in parallel). However, we see that this is not the case in Figure 6D, where different logic operations are performed when both L1 and L2 are included compared to when they are considered alone. In this case, the two operations interact.

In Figure 6E, unlike in Figures 6A,C, the goals (except G^*^, which is always active) send orchestrating feedback, which targets intermediate units in the network. This allows Figure 6E to demonstrate a more complex stimulus-response situation with four different goals (tasks) that the system needs to switch between. These are: G1, to pull lever L1 when cue C1 is presented and L2 when C2 is; G2, to pull L2 for C1 and L1 for C2; G3, to pull L1 for C1 and L1 for C2; and G4, to pull L2 for C1 and L2 for C2. It is due to the orchestrating feedback, which modifies the way the network maps its sensory inputs to its behavioral outputs, that the network is able to perform each of these tasks (Figure 6F).

### 3.3. Phase-dependent operations

**Figure 7:**
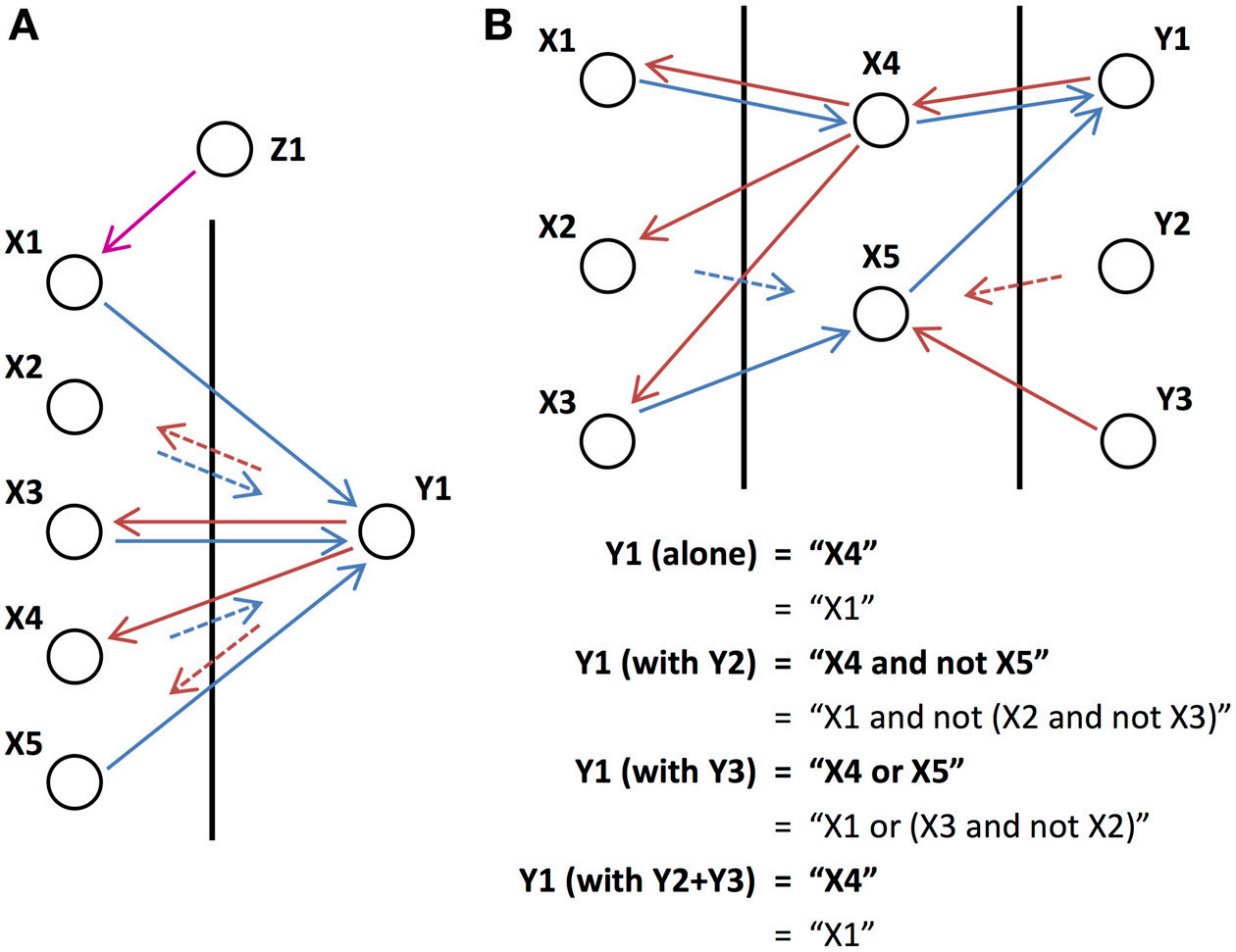
**Phase-dependent operations. (A)** An output unit connected to the five input units, X1-X5, which receive orchestrating feedback from unit Z1 that is out-of-phase with the inputs, performs the operation “(X1 or X3) and not X4” when it is out-of-phase with the inputs and “X3 and not X1 and not X5” when it is in phase with the inputs. **(B)** The operation performed by output unit Y1 is changed depending on which other output (or external) units are initiated with it. Intermediate unit X5 is added by either Y2 or Y3 but in different ways, causing it to play a different role in the operation of Y1, and to perform different operations on its own inputs (X2 and X3).

We found that the relative phase between the output units and the units to which they send feedback can also affect the computations performed. If a network has feedback connections with a mix of short and long time lags and all the inputs are in phase, then some of the units receiving feedback will be activated and others blocked. Figure 7A shows this situation, where switching the phase of the feedback switches which of the input units are activated and which are blocked. It, therefore, modifies the set of inputs that are used in the operation. For example, if Y1 is initiated in phase with the inputs, then only X2 and X5 will be involved in the operation (“X2 and not X5”). But, if Y1 is initiated out-of-phase with the inputs, then only X3 and X4 will be included (“X3 and not X4”). Inputs, such as X1, are persistent and involved in the operation regardless of the phase if they do not receive the feedback from the output units but instead feedback from another source (Z1 in this case).

Unlike gain modulation models where units typically do not have phase, we discovered that, rather than simply adding a unit to an operation (increasing its gain), units can be added with different phases and play a different role in the network. This is shown in Figure 7B, where Y2 and Y3 each send feedback of a different phase to an intermediate unit, X5, adding it to the operation performed by Y1. While both send feedback that adds X5 to the operation of Y1, the different phases of the feedback cause X5 to play a different role in the operation of Y1. With feedback from Y2, X5 is initiated in phase with Y1 and so, due to its long feedforward connection, the operation of Y1 becomes “X4 and not X5.” With feedback from Y3, X5 is initiated out-of-phase with Y1 and the operation of Y1 instead becomes “X4 or X5.” In turn, the different phased feedback causes X5 to perform different operations on its own inputs (X2 and X3). In this situation, we are only concerned with the operation of Y1. However, Y2 and Y3, in addition to modifying the operation of Y1 may also be performing their own operations with their own sets of inputs but these are not shown.

### 3.4. The role of network properties

We investigated how the properties of the feedforward and feedback connections in the network determine the extent to which interactions occur and the operations can be orchestrated. For example, the network in Figure 4B is the same as the one in Figure 4A but with an additional feedforward connection. While this does not affect the operations when they are initiated separately, this additional feedforward connection changes the operations when they are performed together: the input X1 is added to the operation performed by Y2. Figure 4C also performs the same operations as Figures 4A,B when they are performed separately, provided that the feedback to Y2 is in phase with the inputs. However, due to a feedback connection from Y2 to X1, the operations interact when they are initiated together: the input X1 is removed from Y1's operation. Similar to feedback from initiating another output unit, orchestrating feedback from Z1 in Figure 5A modifies the operation of Y1 by adding X1 and removing X2. Adding and removing units is analogous to strengthening or weakening inputs using gain modulation. However, in our model, as shown in Figure 5B, a unit can be added with feedback of a different phase causing it to play a different role in the operation that it is added to. We quantitatively explored how these interaction effects depend on different network connections probabilities in large networks.

#### 3.4.1. Quantifying top-down effects

We quantified the effect of top-down influences by considering the number of inputs that feedback adds or removes from operations of each of the two possible phases. The feedback may be either from other outputs that are initiated or it may be external, orchestrating feedback. No inputs will be added or removed if, and only if, no other operations interact with the operations and orchestrating feedback does not modify the operation. We considered a two-layer network with *N_I_* input units. We defined the following two-component vectors:

- **N_R0_** whose components are the number of input units of each phase that are involved in the computation performed by an output unit (the number of *relevant inputs* of each phase in an operation) when it is initiated without any other outputs initiated or any external feedback.
- **N_R+_** whose components are the number of input units of each phase that are *added* to the set of relevant inputs in an operation when another output unit is initiated or orchestrating feedback from an external unit is present.
- **N_R−_** whose components are the number of input units of each phase that are *removed* from the set of relevant inputs in an operation when another output unit is initiated or orchestrating feedback from an external unit is present.

In order to understand how network properties affect these metrics, we considered a two-layer network with only feedforward and feedback connections (no lateral connections). The connection probabilities for feedforward and feedback connections is *p*_ff_ = *p*_ff−only_ + *p*_ff+fb_ and *p*_fb_ = *p*_fb−only_ + *p*_ff+fb_, respectively, where *p*_ff−only_, *p*_fb−only_, and *p*_ff+fb_ are the probabilities that pairs of units in each layer are connected with only feedforward, only feedback, and both feedforward and feedback connections, respectively. The probability of a feedforward or feedback connection having a long (short) time lag is given by *p_F_* ($p_{\hat{F}}$ = 1 − *p_F_*) and *p_B_* (*p*_${}_{\hat{B}}$_ = 1 − *p_B_*), respectively. The connection probabilities for the feedback from other output units or from external units is given by p^*^_fb_ and the probability of them being long (short) is p^*^_B_ (p^*^_${}_{\hat{B}}$_ = 1 − p^*^_B_). For this situation, we determined the expressions,

(8) $$\begin{array}{l} \frac{N_{R0}}{N_{I}}=p_{ff+\text{fb}}[\alpha ,(1-\alpha )],\\ \frac{N_{R+}}{N_{I}}=p_{\text{fb}}^{*}p_{\text{ff}-\text{only}}[\alpha^{*},(1-\alpha^{*})],\\ \frac{N_{R-}}{N_{I}}=p_{\text{fb}}^{{}^{*}}p_{\text{ff}+\text{fb}}[\alpha (1-\alpha^{*}),(1-\alpha )\alpha^{*}], \end{array}$$

where

(9) $$\begin{array}{l} \;\alpha =1-p_{\bar{B}},\\ \alpha^{*}=\beta \left(1-p_{\bar{B}}^{*}\right)+\left(1-\beta \right)p_{\bar{B}}^{*}, \end{array}$$

and β is the probability that the unit providing the additional feedback is in phase with the output unit being considered. The values α and α^*^ are the probabilities that feedback from the output unit or the other source of feedback, respectively, arrives with the same phase as the main output unit. It is only the time lag of the feedback connection that affects α; however, α^*^ also depends on the likelihood of the other output source being in or out of phase with the main output unit (i.e., β).

**Figure 8:**
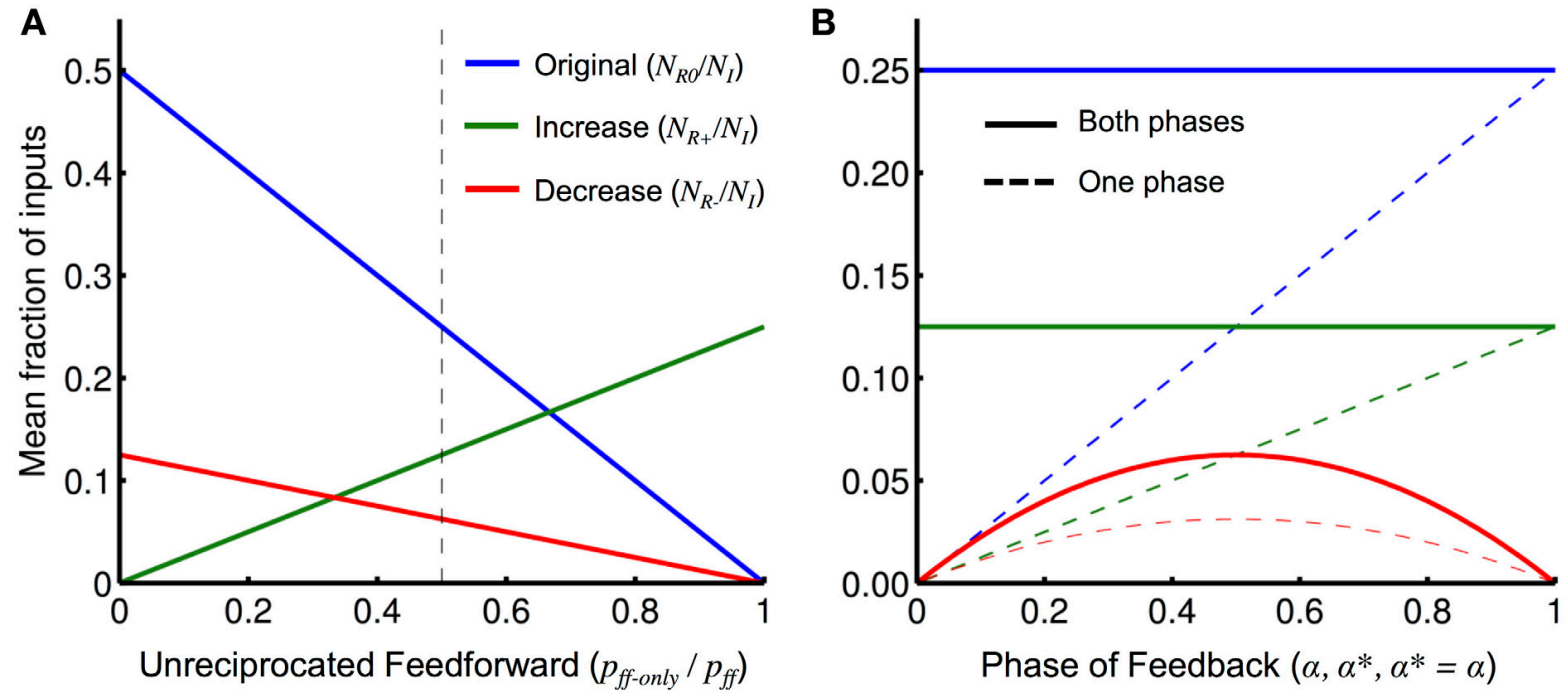
**Interaction effects with network parameters. (A)** The mean fraction of relevant inputs (either phase) for an operation initiated alone, **N_R0_**/*N_I_* (blue), and the mean increase and decrease in the fraction of relevant inputs (either phase) when feedback from a second operation or external unit is also present, **N_R+_**/*N_I_* (green) and **N_R−_**/*N_I_* (red), respectively, plotted as functions of the fraction *p*_ff−only_/*p*_ff_ (the fraction of unreciprocated feedforward connections) as given by Equation (8). The values of other network parameters used were: *p*_ff_ = 0.5, *p*_fb_ = p^*^_fb_ = 0.5, and α = α^*^ = 0.5. The dashed vertical line shows the fraction of *p*_ff−only_/*p*_ff_ used in **(B)**. **(B)** Same as **(A)** but varying the probability of the phase of the different types of feedback: α (phase probability of initiating feedback), α^*^ (phase probability of orchestrating feedback), and α = α^*^ (phase probability of any external feedback), for **N_R0_**/*N_I_*, **N_R+_**/*N_I_*, and **N_R−_**/*N_I_*, respectively. Also shown is the fraction of relevant inputs of a particular phase (dashed) that, compared to the fraction of relevant inputs of either phase (solid), illustrates the split between the two phases.

When the output unit of interest is initiated alone (**N_R0_**), only reciprocally connected units where the feedback is in phase with the inputs will be involved in the operation. Only input units which are not reciprocally connected but make a feedforward connection to the output unit can be added (**N_R+_**) and they are added by receiving feedback that is in phase. This is shown in Figure 8A, where we plot the total number of units (of either phase) originally in the operation and the total number added and removed as functions of the ratio *p*_ff−only_/*p*_ff_. As expected, when there are only reciprocal connections (i.e., *p*_ff−only_/*p*_ff_ = 0), no units can be added; when there are no reciprocal connections (i.e., *p*_ff−only_/*p*_ff_ = 1), no units are originally in the operation (and so none can be removed either).

For a unit to be removed (**N_R−_**), it must originally be in the operation and then receive new feedback that is out-of-phase. This is shown in Figure 8B, where we plot the original number of units in the operation and the number of units that are added and removed for different feedback phase probabilities. For the original number of inputs and the number of inputs added, the total number of inputs is fixed but the split between the phases changes linearly with the value of α and α^*^, respectively. The total number of input units removed is zero when both α = α^*^ = 0 or 1. This is because both types of feedback (from the output unit or the other unit) always arrives with the same phase and so input cannot receive incoherent feedback. Similarly, Equation (9) shows that if α = 0 and α^*^ = 1, or α = 1 and α^*^ = 0, then feedback from the two sources will always be out of phase. In this case, input units of only one phase would be involved in the operation originally and those that receive additional feedback will always be removed.

From Figure 8, we see that there are separate network properties controlling the number of units that are added (*p*_ff−only_/*p*_ff_) and the network property controlling the number of units that are removed (α and α^*^). However, in a random network, it would not be possible to have a mix of phases in the original input units or in the input units that were added and also avoid having units removed from operations.

#### 3.4.2. Interactions and shared inputs

In the random networks we consider, there will be some overlap between the inputs that comprise the operations of different output units but this will not depend on network properties except the likelihood of reciprocal connections and the number of input units. Whether shared or non-shared inputs are added or removed from the operations due to interactions depends on the types of connections involved. Shared inputs are added by interactions due to feedback from reciprocal connections, whereas feedback without a reciprocal feedforward connection adds non-shared inputs. However, input units removed by interactions will always be non-shared as the second output unit must make a interfering feedback connection to the unit.

#### 3.4.3. Interactions in orchestrated networks

Orchestrated networks provide much flexibility for networks to be modified to perform arbitrary operations and this control through high-level, external feedback is a commonly envisioned architecture for gain modulated networks. There are two possible extremes for these types of networks. The first extreme is networks in which many non-interacting operations are performed in parallel. The second are networks in which a larger number of output unit combinations interact to perform a single but potentially more complicated operation. In these two cases, the orchestrating feedback controls and modifies the operations or single operation, respectively.

Considering the first type of network, we investigated the constraints on orchestrating the network if there are to be no interactions or if interactions are to be restricted in some way. An interesting result, shown by Figure 8B, is that *p*_ff−only_ > 0 is required in order to allow external feedback to add additional units to operations but, as long as *p*_fb_ > 0, this means that similar interactions will occur between the operations [see Equation (9)]. This suggests the first of the following three possible network conditions that can control the interactions:

- **No internal feedback:** In this case (*p*_fb_ = 0), operations would require external feedback in order to exist (feedforward activity would not be able to propagate without external feedback) but no interactions would be possible due to the fact that output units could not influence lower-level units at all.
- **Homogenous delays for internal feedback:** In this case, all of the internal feedback would have “short” or “long” delays but not a mix of the two (α = 0 or 1). This means that interactions could cause units to be added to the operations of other units but not removed. Provided that units performed operations that contained at least one unit when they are initiated separately, this network condition would also ensure that if there were no interactions without orchestrating feedback then the addition of arbitrary orchestrating feedback would not change this.
- **Homogenous delays for internal feedback and no non-reciprocal feedforward connections:** In this case (α = 0 or 1, and *p*_ff−only_ = 0), no interactions between output units would be possible and orchestrating feedback would only be able to remove units from operations.

### 3.5. Robustness to noise

Neural oscillations are observed in local field potentials (LFPs) (Roelfsema et al., 1997), which correspond to oscillations and synchrony in the activity of populations of neurons (Gray et al., 1989; Jia et al., 2013). However, Burns et al. (2011) observed that, rather than providing a clock-like signal, gamma oscillations exhibited in LFPs are only auto-coherent for between 2 and 5 cycles. We explored how robust our model is to the introduction of noise to the gamma oscillations.

In the model, input oscillations are represented by activity on alternating time-steps. We model noise by introducing stochasticity to these input oscillations. Instead of the probability of activity on each time-step being 0 and 1 on alternating time-steps, we model noise by either making these θ and 1−θ, respectively, or 0 and 1−θ, respectively, where 0 ≤ θ ≤ 1 is the amount of noise present. These are referred to as “simple noise” and “peak-only noise,” respectively. The second of these ensures that activity never occurs on consecutive time-steps even though noise has been added.

As described by Equations (2) and (4), activation of a unit requires the presence and absence of both feedforward and feedback input activity on specific time-steps. Without noise, these conditions are either fulfilled or not. However, when noise is present, we can analytically derive the likelihood that all of these conditions are fulfilled and the output unit is activated. In any of the networks considered, feedback must activate the apical compartment of the output unit for at least two oscillations: one to put the unit into a searching state and initiate activity in the input units, and one to match the returning feedforward activity and activate the unit. This requires the presence of feedback activity on two time-steps (two apart), the absence of feedback activity on the time-steps preceding each of these, and also the absence of activity two time-steps earlier. Modeling with “simple noise,” these five conditions are satisfied with a probability of (1−θ)^5^, while with “peak-only noise,” the absence of activity on the correct time-steps is guaranteed and the probability becomes (1−θ)^2^. Using a similar approach, the probabilities for the feedforward inputs that lead to activation of the output unit in different networks can be considered. Table 1 shows these feedforward and feedback probability functions when the noise is present in both feedforward and feedback inputs and when it is only present in one of these, and for “simple” and “peak-only” noise. The product of each of the two probabilities gives the probability that the output unit is activated.

**Table 1:** **Derived functions for robustness of different networks to different types of noise**.

**Network**	**Location of noise**	**Type of noise**	**Probability of required feedback activity**	**Probability of required feedforward activity**
OR (2 inputs)	FF+FB	Simple	(1 − θ)^5^	1 − [1 − (1 − θ)^3^]^2^
		Peak-only	(1 − θ)^2^	1 − θ^2^
	FB-only	Simple	(1 − θ)^5^	1
		Peak-only	(1 − θ)^2^	1
	FF-only	Simple	1	1 − [1 − (1 − θ)^3^]^2^
		Peak-only	1	1 − θ^2^
OR (1 input)	FF+FB	Simple	(1 − θ)^5^	1 − [1 − (1 − θ)^3^][1 − θ(1 − θ)^2^]
		Peak-only	(1 − θ)^2^	1 − θ
	FB-only	Simple	(1 − θ)^5^	1
		Peak-only	(1 − θ)^2^	1
	FF-only	Simple	1	1 − [1 − (1 − θ)^3^][1 − θ(1 − θ)^2^]
		Peak-only	1	1 − θ
AND NOT	FF+FB	Simple	(1 − θ)^5^	(1 − θ)^3^[1 − θ(1 − θ)^2^]^2^
		Peak-only	(1 − θ)^2^	1 − θ
	FB-only	Simple	(1 − θ)^5^	1
		Peak-only	(1 − θ)^2^	1
	FF-only	Simple	1	(1 − θ)^3^[1 − θ(1 − θ)^2^]^2^
		Peak-only	1	1 − θ

The networks considered are the “OR” network (with either one or both input units receiving feedforward activity) and the “AND NOT” network (with both input units receiving feedforward activity), while analogous expressions could not be found for the “AND” network. Noise is present in both feedforward and feedback inputs to the network (FF+FB), only feedback inputs (FB-only), or only feedforward inputs (FF-only). The noise is modeled either as “simple noise” or “peak-only noise.”

**Figure 9:**
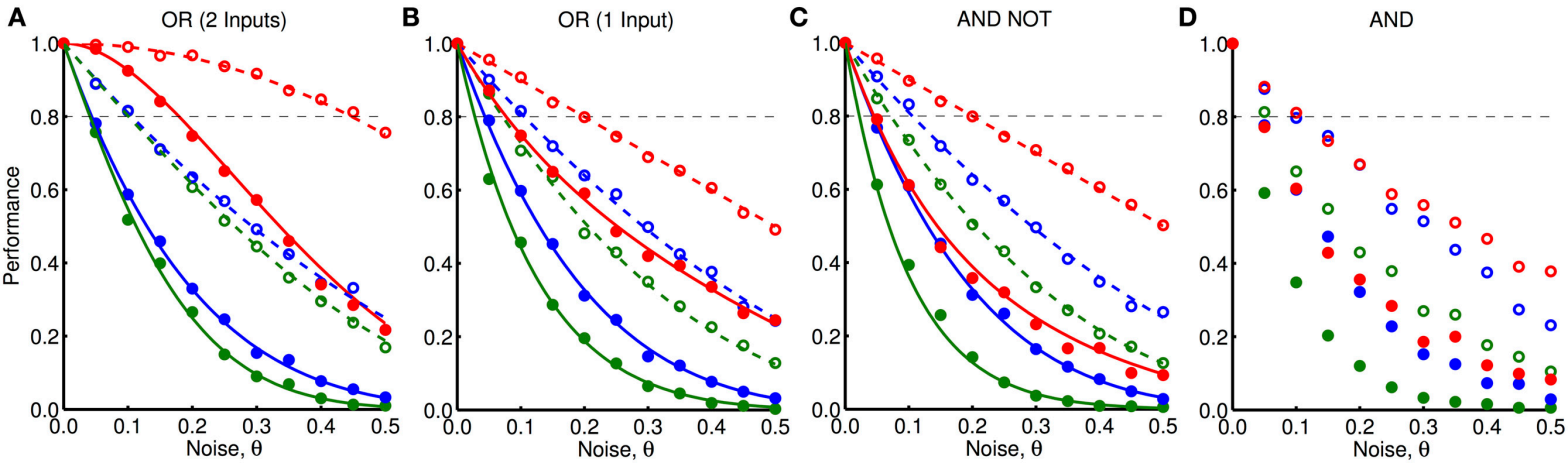
**Robustness to noisy oscillations. (A)** Performance, given by the fraction of trials in which the output unit was activated, of the “OR” network shown in Figure 3 (with both inputs active) to different amounts and types of noise, θ. These different types are: noise only for feedback inputs (blue), noise only for feedforward inputs (red), and noise for both feedforward and feedback inputs (green). The noise can be either “simple noise” (solid) or “peak-only noise” (dashed). Markers show the average outcome from 1000 simulations and lines show analytically derived curves. **(B)** Same as **(A)** but where one input is active and one is inactive. **(C)** Same as **(A)** but for the “AND NOT” network shown in Figure 3 where one input is active and one is inactive. **(D)** Same as **(A)** but for the “AND” network shown in Figure 3 where both inputs are active and without the analytically derived lines.

In Figures 9A,B, we explore the robustness to noise of the “OR” network when both input units receive feedforward activity and when only one input unit receives feedforward activity, respectively. In Figures 9C,D, we explore the robustness to noise of the “AND NOT” and “AND” networks, respectively, when both input units receive feedforward activity. We plot the fraction of trials (out of 1000) in which the output units in each of these networks were activated for different amounts and types of noise (shown by markers). For each of these networks (except for the more complex “AND” network), we compared these simulation results to analytically derived functions for the probability of activation of the output units (shown by lines). For “simple noise,” when θ = 0.5, no oscillations are present (i.e., the likelihood of activity in any time-step is the same) and very poor performance is observed. This highlights the importance oscillations play in these networks. For noise levels of θ = 0.1, the performance dropped down to as low as 40%. However, we observed that “simple noise” impairs the performance of the networks much more than “peak-only noise” in each of the different networks. “Peak-only noise” where θ < 0.05 was sufficient to ensure activation at least 80% of the time (shown by black, dashed lines).

## 4. Discussion

### 4.1. Relation to cognitive phenomena

The networks of cortical units that we have proposed and investigated provides a high-level model of various cognitive phenomena, including goal-directed behavior and top-down attention. We described a general architecture for goal-directed behavior in Figure 2 and demonstrated simple examples in Figure 6. We considered it out of the scope of this study to explore how these goals are generated, maintained, or changed; however, we considered how feedback from goals could quickly switch and modify stimulus-response mappings. This is crucial in behavioral settings, where goals or information held in working memory need to influence the way that stimuli are responded to. In this study, we have identified the different ways that this influence can be implemented. We proposed a model with oscillatory, gain-modulated units that allows feedback to more flexibly manipulate stimulus-response mappings than models with only gain modulation.

Top-down attention naturally arises in this situation because units and subnetworks of units are only activated if they receive feedback corresponding to attention. Stimuli that are not relevant to a particular task will be ignored and activity they elicit will not propagate to higher brain regions. Therefore, bottom-up attention must work via a different means to those described in this study, so that salient stimuli can interrupt top-down tasks and perhaps alter these tasks or goals.

### 4.2. Relation to experimental findings

#### 4.2.1. Context-dependent changes to neural responses

Womelsdorf et al. (2006, 2008) observed context-dependent changes to the receptive field of neurons in the middle temporal area (MT). The changes included shifts of the centers of the receptive fields toward the focus of attention and narrowings of the receptive fields. Cohen and Newsome (2008) similarly observed that noise correlations of MT neurons depended on the current behavioral task being performed. In both of these experiments, the stimuli were not being changed and, according to our model, these context dependent changes are due to changes in the top-down feedback (either initiating or orchestrating) to these neurons.

In low-level areas of the auditory cortex, Zion Golumbic et al. (2013) observed that attention boosted the activity corresponding to “attended speech,” but that “ignored speech” remained represented. However, in higher-order regions, attention becomes more “selective” and activity representing ignored speech was not present. Similarly, in the visual system, Hupé et al. (1998) showed that feedback connections serve to amplify and focus activity of neurons in lower-order areas and that they were important in discriminating between a figure and the background. Schroeder et al. (2010) refer to this interaction between sensory and attentional, top-down signals as “active sensing.” This is consistent with the model we are proposing where attention, determined by the goals of the system, “selects” the relevant sensory stimuli, while ignoring irrelevant stimuli.

#### 4.2.2. Abstract rules and operations

Wallis and Miller (2003), Muhammad et al. (2006), and Buschman et al. (2012) considered abstract rules that could be applied in a very similar manner to many different stimuli-response mappings. The ability of the brain to create such abstract mappings suggests that it reuses the same circuitries or networks for multiple analogous purposes. This is consistent with the way networks in our model can be composed together and embedded into larger networks. In this case, it is the role of orchestrating feedback to make sure the reused network receives the appropriate inputs and that its outputs are used correctly. Badre (2008) reviewed the evidence for hierarchies within goals and rules used for cognitive control in the PFC where there were increasing levels of abstraction for higher-level goals. This hierarchy of goals suggests the existence of different levels of goal-dependent feedback, each orchestrating different parts of the stimulus-response mapping required for the over-arching goal.

Buschman et al. (2012) showed that during a stimulus-response task there was a dominant rule (based on the orientation of a visual stimulus), which alpha oscillations appeared to suppress in order for a different rule (based on the color of a visual stimulus) to be employed. In our model, this type of behavior may be exhibited by having orchestrating feedback that would modify the original, dominant operation or mapping performed by the network to a secondary mapping. However, our model does not suggest an explanation as to why alpha rhythms would be involved in this top-down remapping.

#### 4.2.3. Fast signal propagation and neural coding

In our model, activity takes at most half the oscillation period (about 7–10 ms for gamma oscillations) to propagate from one unit to the next. The target unit does not need to integrate its inputs but can very quickly pass along the “signal” provided that it receives coherent feedforward and feedback inputs. In other words, units in the model are assumed to exist in a fluctuation-driven regime, where, unlike models in which units need to integrate their inputs, activity can be more rapidly altered. This is consistent with the range of reaction times (about 300–400 ms) observed by Wallis and Miller (2003) in their rule-based behavioral experiments. In our model, both the phases and the levels of activation (absolute firing rate) are important for performing computations. Our model does not predict that absolute spike rates are irrelevant but it does make the assumption that they are only relevant in concert with the appropriate phases.

#### 4.2.4. Pyramidal neurons

Larkum et al. (1999, 2001, 2004) and Larkum (2013) observed that pyramidal neurons exhibited a much stronger response when they received inputs both to their soma (and basal dendrites) and to their apical dendrites than they did when they received only one of these types of inputs. In addition to the spike initiation zone at the cell body for action potentials (sodium spikes), there is a second initiation zone near the apical tuft of layer 5 pyramidal neurons (Yuste et al., 1994; Schiller et al., 1997; Larkum and Zhu, 2002). This second initiation zone produces broad calcium spikes within the cell and its existence suggests that pyramidal neurons should be considered to have two functional compartments. Larkum et al. (1999, 2001, 2004) and Larkum (2013) discuss how interactions between these two initiation zones, where spikes from either one lower the firing threshold of the other, provide the associative mechanism whereby a stronger response occurs when both somatic and apical inputs are present.

We proposed our analogous model for interconnected groups of pyramidal neurons based on this experimentally-based description of how pyramidal neurons respond to different types of inputs. In our model, groups of neurons behave similarly to individual pyramidal neurons in that they produce a much stronger response when receiving somatic feedforward activity as well as apical feedback. However, our model differs in that the groups of neurons also contain inhibitory interneurons and because of this they exhibit oscillatory activity in the gamma frequency range.

### 4.3. Experimental predictions

#### 4.3.1. Requirements of neural activation

In our model, the activation of groups/ensembles of neurons requires strong coherent feedforward and feedback activity. We are predicting that, at least during goal-directed tasks, neuronal ensembles in high-level areas of the cortex are only activated if they receive feedback from higher regions, or if there has been recent feedback (regions involved in working memory may sustain activity without feedback). Similarly, without receiving activity from lower-level units, our model predicts that high-level units would at most be able to exhibit sporadic, searching activity and not strong oscillatory (e.g., gamma frequency) activity.

This prediction does not necessarily extend to lower-level areas of the cortex, such as V1, in which sensory input alone may be sufficient to activate groups of neurons (Hupé et al., 1998). There may also be top-down feedback present during non-goal-driven behavior, or during resting states, that provides a “default” set of operations for the network. Similar to this, the presence of a neuromodulator may remove (or introduce) the need for top-down feedback, allowing feedforward activity alone to activate units and propagate into higher-level regions. For example, there is evidence that cholinergic neurons increase the amount that attention modulates the activity of cortical neurons (Herrero et al., 2008; Goard and Dan, 2009; Thiele, 2009; Herrero et al., 2013). In this situation, acetylcholine may actually decrease the excitability of the neurons, pushing them into a more goal-driven mode, where they are forced to rely on both feedforward and feedback activity to become active.

We hypothesize that there must also exist coherence between neurons within an active ensemble and between neurons in different active ensembles that are strongly connected. This prediction is most relevant to the activation of neuronal ensembles during attentional and behavioral tasks. This type of experimental result has been observed for alpha and beta frequencies by Buschman et al. (2012), where there was coherence between neurons in the PFC during behavioral tasks that involved switching between different abstract rules.

In addition to coherence, we predict that, at a network level, activation also requires periodic or oscillatory activity. The reason for this is demonstrated in the first circuit of Figure 3 (“X1 or X2”). Here, feedback arrives at Y1, propagates to X1 one time step later, and, if this coincides with feedforward input to X1, feedforward activity travels back to Y1 another time step later. This means that the returning feedforward activity arrives back at Y1 one period of oscillation later and Y1 only becomes activated if the feedback it received was oscillatory, where another round of synchronous feedback coincides with the delayed, returning feedforward activity. In this way, oscillations do not simply allow persistent activation, in many cases, they are necessary for the output units of subnetworks to become active at all.

#### 4.3.2. Robustness to noise

As shown in Figure 9, simple networks of our cortical unit model are sensitive to noise in their oscillatory inputs. For realistic noise levels (θ of 10%) the performance drops to approximately 40–50% with “simple noise” or 60–80% with “peak-only noise.” Furthermore, this sensitivity would, in most cases, be amplified for larger networks with more sophisticated operations. For this reason, it is critical that future studies explore more detailed and biologically realistic models of noise in neural oscillations and explore possible mechanisms for models to cope with this noise. This would lend biological plausibility to our present model, which is currently not sufficiently robust to noise. One mechanism that would help improve the robustness to noise would be to have a redundant architecture, where multiple networks perform the same operation but receive independent noise.

Figure 9 also showed that simple operations were much more robust to “peak-only noise” than to “simple noise.” This was because “peak-only noise,” unlike “simple noise,” can only lead to absence of activity when it was expected and not the presence of activity on the other time-steps. Although “simple noise” provides a good model of noise for individual neurons, “peak-only noise” may potentially be a more realistic model for the groups of excitatory and inhibitory neurons that we are modeling. While activity may fail to be initiated on a given oscillation cycle within a group of neurons, the inhibitory activity following such neural activity should reliably suppress further activity for a short period of time. This fits the “peak-only noise” model in which sustained activity (activity on consecutive time-steps) is not permitted to occur.

Another more detailed model of oscillation noise would be one in which time is modeled as being continuous and the oscillation period is modeled as stochastic. This would align more closely with experimental observations, such as those by Burns et al. (2011). It would also mean that, while the oscillations may not be auto-coherent over more than a few cycles, the time between any two consecutive peaks would only vary slightly. Given that connection delays in our model are at most half the oscillation period, it is only the auto-coherence over one period that would be of importance. We speculate that a model similar to ours with continuous time (and therefore continuous phases) would be very robust to noise modeled in this manner and this robustness would likely scale well to larger networks. However, this would depend on how closely input phases would be required to align in order for units to be activated.

#### 4.3.3. Different cortical regions

The results from Equation (9) and Figure 8 for large networks suggest a trade-off between the ability to perform many operations in parallel and the ability to control these operations in a top-down manner with feedback. Given that different regions of the cortex would have different priorities in this regard, this makes experimental predictions for the connectivity within and between different regions of the cortex. For instance, within regions where units would correspond to percepts, interactions between hypotheses and goal-directed manipulation of hypotheses would be expected to be low as our perceptions are relatively stable with respect to our goals. In this case, we would expect that α^*^ ≈ 1, *p*_ff−only_ ≈ 0, and p^*^_fb_ ≈ 0. In other words, feedback connections from units in one hierarchical level to another would be expected to have quite similar propagation delays, feedforward and feedback connections between units would mostly occur together (i.e., reciprocally), and there would be few feedback connections coming directly from regions involved in motivations/goals/behavior. For higher-level regions where units correspond to actions and more abstract concepts, goal-directed orchestration of operations, and perhaps also interactions between different operations, would be desired. For the regions involved in motivation and goals to orchestrate the operations and control the network, there needs to be feedback connections from these regions to the units (i.e., p^*^_fb_ > 0).

**Figure 10:**
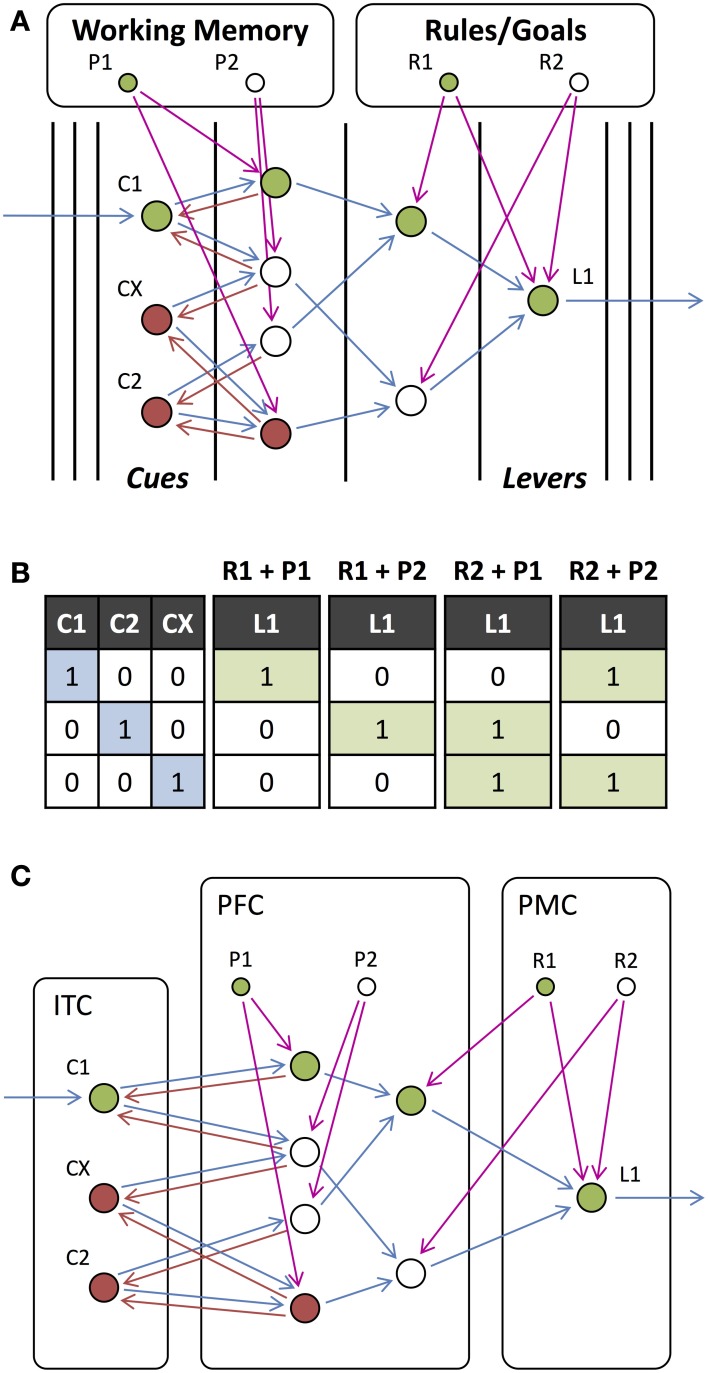
**Stimulus-matching Experiment. (A)** Network for responding to a stimulus (C1, C2 or any other stimulus CX), which is dependent on the current rule that determines whether to respond to a match or non-match with the previous stimulus. In contrast to previous tasks, working memory is required to remember the previous stimulus (P1 and P2 which correspond to the same stimulus as C1 and C2, respectively) in the same way that the rules (R1 and R2) are remembered. **(B)** Input-output table for the network presented with a stimulus for combinations of rules and remembered stimuli. **(C)** Same network as in **(A)** but with units assigned to anatomical regions of the cortex.

Wallis and Miller (2003) and Muhammad et al. (2006) recorded from neurons in the prefrontal (PRC), premotor (PMC), and inferior temporal (ITC) cortices and the striatum (STR) during a stimulus-matching task. During the task, two visual stimuli were presented and, depending on the rule (which was indicated via a visual cue presented with the first stimulus), the subject was required to either continue holding a lever or release the lever. They observed different neurons that responded selectively to the rule (desired stimulus-response mapping), the behavioral response carried out, the visual stimulus being remembered, or whether the subsequent stimulus matched this remembered stimulus. We constructed a possible network to carry out this task (Figures 10A,B). In addition to external feedback that depends on the rule to be employed, this network receives external feedback based on the stimulus being remembered (held in working memory). Based on the selectivity that was observed of neurons in different cortical regions, we divided this network into these different regions (Figure 10C). While this is not necessarily the exact network used for this task or the correct allocation of units to cortical regions, this demonstrates how our model may be useful in understanding the role of neurons in different regions.

#### 4.3.4. Searching feedback and neuronal avalanches

The sporadic, bursting feedback activity that we proposed to be exhibited by units during the searching state is based on the observations by Larkum (2013) of the activity of pyramidal neurons that receive only strong input to their apical dendrites. We propose that this mechanism exists for ensembles of pyramidal neurons and that it is used to pass internal predictions/expectations from higher-level ensembles down to lower-level ensembles. This relies on sharp, sporadic, bursts of activity being able to propagate along feedback connections down to lower-levels but not along feedforward connections up to higher-levels. Neuronal avalanches observed *in vitro* (Beggs and Plenz, 2003) may correspond to spontaneous examples of these searching signals within networks that are not receiving any sensory inputs. Of interest would be the calcium activity, due to the activation of the calcium spike initiation zone on the apical branch, of pyramidal neurons during neuronal avalanches.

#### 4.3.5. Apical-targeted feedforward connections

We have assumed that higher level areas connect to lower level areas via feedback (apical-targeted) connections and that, in return, the lower level areas connect to the higher level areas via feedforward (soma/basal-targeted) connections. While this seems to be the prevalent pattern in the cortex, exceptions are likely to exist. Apical-targeted feedforward connections, for example, would allow salient, lower-level features/hypotheses to prompt/pose higher level hypotheses. The opposite (soma/basal-targeted feedback connections) may also exist but it is not clear the functional role that these connections could play.

### 4.4. Neuronal ensembles and lateral connections

Units in our model are not necessarily local or spatially distinct groups of neurons, but are instead defined by their functional connectivities. In other words, units are better thought of as strongly interconnected ensembles of neurons. We have assumed, in this study, that units consist of distinct, non-overlapping groups of neurons. It may be, however, that there is a large overlap between the neurons that make up each unit.

We considered feedforward and feedback connections between units but not lateral connections. In this situation, feedforward activity depends on feedback activity but feedback activity does not depend on feedforward activity as it can only determine whether units are in “searching” or “active” states [see Equation (2)]. This means that there are no “loops” present in networks that can lead to unstable dynamics. Lateral connections add considerable complexity as they potentially introduce these “loops.” However, assuming that units in the same layer consist of overlapping groups of neurons, it may be better to think of lateral connections between units as interactions between these overlapping ensembles, in which the ensembles either inhibit each other (both cannot be active: winner-take-all), reinforce each other (performing an “OR” operation), or require co-activation (performing an “AND” operation). In the situation, where units laterally inhibit each other, the hypotheses they represent are incompatible. This is similar to multistable perceptual phenomena, such as binocular and monocular rivalry, where there is competition between two incompatible perceptions. Leopold and Logothetis (1999) showed that, during binocular rivalry experiments, a greater number of neurons in higher-level areas are correlated with the perception than in lower-level areas, suggestive of top-down processing.

### 4.5. Relation to gain modulation

Previous studies consider gain modulation, caused by feedback signals, as a means of performing top-down processing and cognitive control. In the study by Salinas (2004), fixed feedback dependent on the current rule was used to modulate the gain of neurons with feedforward connections on output neurons. This is functionally similar to the last two layers of the network we considered in Figure 6E, where there are only feedforward connections between the layers and external feedback orchestrates/modulates the inputs to be considered. In contrast to such studies, our model exhibits an exaggerated and simplified example of non-linear gain modulation, where feedback modulates feedforward signals above a threshold, which cannot be otherwise achieved and which permits further propagation of the signal (see Figure 1C). It remains to be explored how our findings, regarding interacting, orchestrated, and phase-dependent operations, extend to the case where activities and connection strengths are continuous. Instead, our model has only binary inputs, states (besides the searching state), and connections. Because of this, our simplified model is not well suited to integrating many individual inputs and determining whether all are present or a threshold has been reached. A more detailed model with continuous activation levels and connection strengths would be more suited to these types of computation. However, we suggest that this type of processing is potentially more prevalent in lower-level regions of the cortex and that, in higher-level, associative areas, it may play a smaller role.

With only gain modulation, feedback can either increase or decrease the gain of a neuron and, therefore, how strongly it is involved in an operation, but it cannot change the role that it plays in the various operations in which it is involved. Here, however, the top-down feedback can have different phases that can initiate units with different phases giving them different roles in operations.

### 4.6. Future extensions

#### 4.6.1. Synaptic plasticity

When investigating how network properties affected top-down processing, we only considered randomly connected networks, whereas when we considered a specific task, the networks we used had a very specific structure. This specific structure would need to emerge due to some form of activity-dependent synaptic plasticity. Spike-timing-dependent plasticity, for instance, would be expected to reinforce connections between active, coherent units (Kerr et al., 2013). It may also be that, in the case where a set of connections only ever inhibits the activity of the target unit, synaptic plasticity only maintains the connections onto the inhibitory neurons that cause this inhibition. This would mean that this set of connections becomes only able to inhibit and not activate the target unit. In addition to this, it remains to be investigated how robust certain networks or motifs are to the introduction (removal) of units to (from) operations through synaptic plasticity. We speculate that networks with fewer interactions may be more robust in this regard but this remains to be explored.

#### 4.6.2. Analysis of networks with three or more levels

Our exploration of network properties only considered the case of a two-layer network of units. We would expect that interactions between different operations would be amplified in a network with more layers because more units and connections, which can cause the interactions, are involved. However, connections do not need to be restricted to being between adjacent layers. For instance, this is not the case in Figures 6, 7B. An analysis of how different network properties affect top-down processing and interactions becomes more complicated in this case.

#### 4.6.3. Other phases and frequencies

We have assumed that active units in our model oscillate at one of only two different phases. This simplified model could be extended to include a continuous range of phases, better capturing the complexity of networks in the brain. More possible phases would increase the number of different operations in which units/ensembles could be used. With more than two phases, activation of units would not require the exact matching of phases but only that the phases are sufficiently similar. In a more detailed model, there may be degrees of activation depending on how similar the incoming phases are. In addition to having many phases, neurons within an ensemble may actually exhibit a range of phases rather than just a single phase. This would further complicate the ways that units affect each other through lateral “connections,” where neurons are shared between units, or actual feedforward and feedback connections, where the phase of the individual source and target neurons differs within the units.

Our model would similarly apply to other inhibitory-based rhythms (Whittington et al., 2000), such as beta frequency oscillations. In fact, there is experimental evidence to suggest that beta frequency oscillations, either alone or interacting with gamma oscillations, may represent a better candidate for top-down modulations (Engel and Fries, 2010; Benchenane et al., 2011). For example, Buschman et al. (2012) showed that neurons in the PFC synchronized to beta frequencies during a rule-based behavioral task.

A number of other roles for gamma oscillations have been proposed based on experimental observations. Schroeder and Lakatos (2009) argued that the amplitude of gamma oscillations is often coupled to the phase of, or “enslaved” to, lower frequency oscillations (e.g., delta or theta) and propose that non-enslaved gamma oscillations are only exhibited during a “vigilance” mode when there is no task relevant rhythm. Arnal et al. (2011) proposed that gamma oscillations represent bottom-up prediction errors, indicating when sensory signals misalign with top-down predictions represented by beta oscillations.

This study focuses on a single spectral band associated with sensory processing and motor pattern generation. However, multiple frequencies are likely present at the same time and future work exploring this situation would be very interesting. For example, there is evidence to suggest that feedback activity would likely be at lower frequencies (e.g., beta) while feedforward activity would be at higher frequencies (e.g., gamma) (Arnal et al., 2011; Bastos et al., 2012). The role of these different frequency oscillations, and how they may interact in situations such as this, while out of the scope of the current study, promises a rich area for exploration. Unless the lower frequencies are subharmonics of the higher frequencies, how different frequency oscillations would interact poses a problem that needs to be investigated. Alternatively, computations with different frequencies could potentially operate in parallel to each other.

#### 4.6.4. Detailed models of neural activation

In this study, we have considered only three discrete activation levels for units of neurons (resting, searching, and active). An area for future work is to consider more detailed models of neural activation in which units have continuous levels of activation. In this case, feedback could be modeled as smoothly (although most likely non-linearly) modulating the sensitivity, or gain, of units to feedforward input, rather than simply gating the activity of units. The conditions under which top-down feedback would play a major role in activating and modifying neural ensembles and the computations that they preform remains to be explored. Another interesting area of investigation would be modeling the effects of neuromodulators, such as acetylcholine, on neural activation and exploring how such neuromodulators could switch a network between bottom-up, forward-driven and top-down, feedback-driven modes of operation.

#### 4.6.5. Phase-locking of top-down oscillations

Feedforward and feedback phases propagate forwards and backwards, respectively, according to connections that have been established through plasticity mechanisms, which remain to be explored in this context. However, there would ultimately be the bottom-most feedforward phases and the top-most feedback phases and, in order for them to synchronize and activate along the appropriate network pathways, some sort of coordination between these phases would be necessary. In order to accomplish this, high-level areas of the brain would need to perform “phase-locking” (as opposed to the “phase-matching” focussed on in this study) between their top-down signals and the bottom-up signals in lower-level areas. This “phase-locking” would occur over the network as a whole and would allow “phase-matching” to perform the operations as described in this study. This type of synchronization must be ubiquitous in the brain and at high frequencies, such as gamma and beta, it should be possible to perform this “phase-locking” quickly. Together with an investigation of a model in which there are continuous phase ranges, future work lies in investigating how this type of “phase-locking” could be carried out between top-down and bottom-up oscillations.

#### 4.6.6. More specific connections

While we separated inputs into two types (apical and basal), each of these could be further split up into individual dendritic branches that locally integrate their own inputs (Larkum et al., 2009) and can be targeted specifically by certain inhibitory inputs (Palmer et al., 2012). Targeted inhibition to specific branches and, therefore, inputs would allow neurons/units to perform much more complicated computations and, in particular, would be useful in allowing neurons to be re-used for different operations. In addition to this, excitatory connections to a unit could specifically target either the excitatory or inhibitory neurons in the unit.

## Author contributions

Robert R. Kerr conceived the study; Robert R. Kerr, David B. Grayden, Doreen A. Thomas, Matthieu Gilson, and Anthony N. Burkitt designed the research; Robert R. Kerr performed the research; Robert R. Kerr, David B. Grayden, Doreen A. Thomas, Matthieu Gilson, and Anthony N. Burkitt wrote the paper.

## Funding

Funding is acknowledged from the Australian Research Council (ARC Discovery Project DP1096699). The Bionics Institute acknowledges the support it receives from the Victorian Government through its Operational Infrastructure Support Program. This work was supported by the Australian Federal and Victorian State Governments and the Australian Research Council through the ICT Centre of Excellence program, National ICT Australia (NICTA). The funders had no role in study design, data collection and analysis, decision to publish, or preparation of the manuscript.

### Conflict of interest statement

The authors declare that the research was conducted in the absence of any commercial or financial relationships that could be construed as a potential conflict of interest.

## Supplementary material

A sample of the code used in this study can be found online at: https://github.com/NeuroEngineering-UniMelb/cortical-units
